# Chagas-specific Death and Competing Risk of Mortality in Chile: A Nationwide Retrospective Cohort Study, 2007–2021

**DOI:** 10.1093/ofid/ofag502

**Published:** 2026-08-14

**Authors:** Franco Fernández-Guardiola, Claudia T Codeço, Natalia Vergara, Jorge Vilches, Diego Sandoval-Vargas, Eduardo A Undurraga, Mauricio Canals, Kasim Allel

**Affiliations:** School of Public Health, Faculty of Medicine, University of Chile, Santiago, Chile; SENTINET: Surveillance, Epidemiology, and New Technologies for Infectious Emerging Threats, Chile, Pontificia Universidad Católica de Chile, Santiago, Chile; Department of Medical Technology, Faculty of Medicine, University of Chile, Santiago, Chile; Scientific Computing Program, Foundation Oswaldo Cruz, Rio de Janeiro, Brazil; Department of Epidemiology, Undersecretariat of Public Health, Ministry of Health of Chile, Santiago, Chile; Department of Epidemiology, Undersecretariat of Public Health, Ministry of Health of Chile, Santiago, Chile; Centre for Biotechnology and Engineering (CEBIB), Department of Chemical Engineering, Biotechnology and Materials, University of Chile, Santiago, Chile; SENTINET: Surveillance, Epidemiology, and New Technologies for Infectious Emerging Threats, Chile, Pontificia Universidad Católica de Chile, Santiago, Chile; School of Government, Pontificia Universidad Católica de Chile, Santiago, Chile; School of Public Health, Faculty of Medicine, University of Chile, Santiago, Chile; SENTINET: Surveillance, Epidemiology, and New Technologies for Infectious Emerging Threats, Chile, Pontificia Universidad Católica de Chile, Santiago, Chile; Nuffield Department of Primary Care Health Sciences, University of Oxford, Oxford, UK; Department of Infectious Diseases, School of Medicine, Pontificia Universidad Católica de Chile, Santiago, Chile

**Keywords:** Chagas disease/epidemiology, Chagas disease/mortality, Chile/epidemiology, competing risks, hospitalization/statistics and numerical data

## Abstract

**Background:**

Chagas disease (CD) in Chile has shifted toward a postvector-control, aging-cohort scenario in which chronic sequelae increasingly shape outcomes. We quantified Chagas-specific mortality and associated factors using competing-risks methods.

**Methods:**

We conducted a nationwide retrospective cohort study of confirmed CD cases notified in Chile during 2007–2021, linked to mortality and hospital discharge records. Chagas-specific death was the primary outcome, with other-cause death as a competing event. We estimated cumulative incidence functions (CIFs), applied Gray's test, and fitted Fine–Gray models for subdistribution hazard ratios (sHRs). Complementary cause-specific Cox models included hospitalization as time-fixed and time-varying markers of clinical severity. Models accounted for the national cohort structure and competing mortality.

**Results:**

Among 17 508 individuals, 261 (1.49%) died from CD and 1021 (5.83%) from other causes. Ten-year CIF was higher in men (2.66%) and increased with age, reaching 4.80% at 65–74 years and 8.89% at ≥75 years. Cumulative incidence functions was highest for chronic digestive disease (B57.3, 6.49%) and elevated for chronic cardiac disease (B57.2, 2.93%) compared with Z22.8 (1.47%; Gray *P* < .005). In adjusted Fine–Gray models, mortality was associated with each 10-year age increase (sHR 2.31), male sex (sHR 1.47), B57.2 (sHR 1.92), and B57.3 (sHR 3.36). Cause-specific Cox models showed a graded association between recurrent hospitalizations and Chagas-specific death (≥3 admissions: HR 83.94), consistent with advanced clinical deterioration.

**Conclusions:**

Although Chagas-specific death was uncommon, it was concentrated among identifiable high-risk groups. Risk-stratified chronic care and structured follow-up should prioritize patients with organ involvement or recurrent hospitalization.

## Teaser

Competing-risks analysis of nationally reported Chagas disease cases in Chile shows that Chagas-specific mortality is low overall but concentrates in older adults, men, and organ-involving forms, especially chronic digestive and cardiac disease. Cause-specific Cox models identify hospitalization, particularly recurrent admissions, as the strongest marker of advanced deterioration and elevated near-term risk of Chagas death.

## Hospitalization

Chagas disease (CD), a parasitic infection caused by the protozoan *Trypanosoma cruzi* [1, 2] affects an estimated 7.5–12 million people worldwide, causing between 10 600–12 500 deaths annually [3, 4]. Chagas disease poses an annual global cost of approximately $627 million in direct health costs, with a total global cost, including productivity losses, estimated at $7.2 billion per year (2012 USD) [5]. The global prevalence of CD has decreased from an estimated 7.3 million to 6.5 million cases (an 11.3% decrease), with the total burden in disability-adjusted life years declining by 23.7% between 1990 and 2019 [6]. However, 96% of global cases remain concentrated in Latin America, driven by persistent social and health-system vulnerabilities such as rural poverty, substandard housing that facilitates triatomine domiciliation, and uneven access to timely diagnosis and treatment [6–8].

As the 2 largest contributors in Latin America, Brazil (2.16 million cases, 33.5% of the global total, prevalence of 912.36 per 100 000), and Argentina (11%–15% of the global infections, prevalence of 1524 cases per 100 000) account for a substantial share of the global CD burden [6]. In Chile, prevalence has increased (1114 cases per 100 000) driven by nonvectorial transmission routes, particularly congenital transmission, which remains an important source of new infections despite the interruption of domestic vectorial transmission [9, 10].

A further concern is the potential resurgence of vector transmission from sylvatic triatomines that are not effectively controlled in rural areas [11, 12]. In Chile, the hospitalization rate for chronic CD decreased by over 70% between 2001 and 2014 [13]. Although Chagas cardiomyopathy is the most clinically relevant manifestation due to its prognostic implications, digestive involvement is the leading cause of hospitalization, accounting for 55.8% of all chronic CD admissions [14, 15]. Moreover, patients with digestive manifestations are substantially more likely to undergo surgical intervention than those with cardiac forms (relative risk 4.4; 95% CI 2.9–6.9), underscoring the substantial healthcare burden of digestive CD [13]. Taken together, these trends suggest evolving clinical and epidemiological patterns in Chile despite overall declines in severe outcomes.

Chile represents a postvector-control, aging cohort setting in which congenital transmission, migration, and chronic cardiac/digestive sequelae increasingly shape outcomes. However, the determinants of Chagas-specific mortality and the contribution of competing causes of death remain poorly characterized. Using linked, population-level data and competing-risks methods, we analyzed mortality among individuals diagnosed with CD to identify demographic and clinical factors associated with Chagas-specific death and to inform risk stratification, follow-up strategies, and program monitoring in Chile and other post-transmission settings. These objectives are consistent with the WHO 2030 NTD roadmap [16], which emphasizes integrated, outcome-oriented care beyond transmission interruption.

## METHODS

### Study Design

We conducted a nationwide retrospective cohort study in Chile to analyze reported and confirmed cases of CD, following the 2014 National Technical Guidelines for the Control and Prevention of CD [17]. The study covered a 15-year period, from 1 January 2007 to 31 December 2021, and included health records from public and private health facilities across all 16 administrative regions of the country.

### Data Sources

Data were collected from national notification records as part of the Chilean mandatory notification system.

We included cases classified as laboratory-confirmed CD according to the national surveillance definitions applicable during the study period. Under Chilean diagnostic guidelines, confirmation of chronic infection is generally based on concordant results from 2 serological assays using different diagnostic principles or antigenic preparations, with discordant results requiring additional evaluation or confirmation by a reference laboratory (see Supplementary Text 1). Diagnostic approaches for suspected acute or congenital infection may additionally include direct parasitological or molecular methods, depending on the clinical and epidemiological context [17]. However, the notification database did not contain patient-level information on the assays performed, test manufacturers, results combinations, confirmatory procedures, or the laboratory responsible for testing. We were therefore unable to reconstruct or independently verify the diagnostic pathway used for each notified case.

These data were linked to mortality and hospital discharge records from Chile's Department of Statistics and Health Information using unique national identifiers, enabling the linkage and harmonization of diagnosis dates, hospital admission and discharge dates, and dates of death across datasets. The final analytical sample included 17 508 cases of CD, averaging 1167 per year between 2007 and 2021. As the study covered 2007–2021, diagnostic technologies, laboratory availability, screening coverage, and confirmatory procedures may have evolved over time. Although all included records met the surveillance definition of a confirmed case, the available data did not allow assessment of temporal or regional variation in the specific diagnostic algorithms applied.

### Primary Outcomes and Independent Variables

The primary outcome was Chagas-specific mortality, defined as death with CD recorded as the underlying cause of death during follow-up. Death from other causes was treated as a competing event. Hospitalization related to CD was considered both a secondary outcome and modeled as a time-varying prognostic marker during follow-up. We collated the dates of confirmed diagnosis and death, whenever applicable. The number of hospitalizations and total length of hospital stay (LOS), measured in days, were also recorded.

Independent variables included biological sex, age at diagnosis, and disease stage at diagnosis (ie, acute or chronic). Clinical presentation was classified using ICD-10 codes as acute CD with cardiac involvement (B57.0), acute CD without cardiac involvement (B57.1), chronic CD with cardiac involvement (B57.2), chronic CD with digestive involvement (B57.3), chronic CD with nervous system involvement (B57.4), chronic CD with other organ involvement (B57.5), and congenital CD identified through codes related to fetal or neonatal exposure to maternal infectious disease (P00.2). The Z22.8 code, corresponding to carrier status for other specified infectious diseases, was used in the Chilean notification records primarily for asymptomatic individuals and infections detected through screening, including blood donation. These classifications were not independently validated against individual clinical records or specialist assessments. Geographic location was defined using macrozones (North, Central, South, and the Metropolitan Region) based on the region where cases were reported. Age was handled according to the purpose of each analysis. For descriptive, incidence, crude mortality proportion, and cumulative incidence analyses, age was stratified into predefined categories (<15, 15–24, 25–34, 35–44, 45–54, 55–64, 65–74, and ≥75 years). The first category distinguished children and younger adolescents, followed by 10-year age bands and an open-ended category for the oldest individuals. For Fine–Gray and cause-specific Cox regression models, age was modeled as a continuous variable per 10-year increase, avoiding information loss from dichotomization.

### Statistical Analysis

We described cases using counts or percentages, medians, and the interquartile range (IQR). We conducted group comparisons between deceased and surviving patients utilizing the χ^2^ test and the Kruskal-Wallis test for LOS. Annual notified incidence was calculated per 100 000 population per year, using mid-year population estimates as denominators. Crude death proportions of Chagas-specific deaths (ICD-10 Chagas as cause of death) were calculated by sex, age group, diagnostic stage, ICD-10 category, and hospitalization status for descriptive purposes. Missingness was assessed separately for each covariate. Descriptive analyses used all available observations, with variable-specific denominators reported where applicable. Regression analyses were restricted to individuals with complete information for all covariates included in each model; no missing values were imputed (see Supplementary Section 2).

We analyzed Chagas-specific mortality, allowing for competing events, with follow-up from notification to the earliest of Chagas-specific death, death from other causes, or administrative censoring (31 December 2021). We estimated cause-specific cumulative incidence functions (CIFs), compared groups using Gray's test, and fitted Fine–Gray subdistribution hazard models (univariable and multivariable) to report adjusted subdistribution hazard ratios (sHRs) for Chagas-specific deaths. As a complementary approach, we fitted cause-specific Cox models for Chagas-specific death (other-cause deaths censored at their time of occurrence), using cluster-robust standard errors at the regional level. Proportional hazards were assessed using Schoenfeld residuals. Final Cox models were stratified by diagnostic stage (acute vs chronic) to allow stage-specific baseline hazard rates. Hospitalizations were examined under time-fixed (ever/number of admissions) and time-varying start-stop specifications (postfirst admission and accumulated admissions), with model comparisons based on AIC and the concordance index.

We report this cohort study in accordance with the STROBE statement. Full methodological details and results of these analyses are provided in the Supplementary Materials. All analyses were conducted in Python (version 3.10) and R (version 4.3.1), using 2-sided *P* values (*α* = .05) for statistical inference. The analysis code is available on GitHub (https://bit.ly/Competing_risks_analysis)

## RESULTS

### Descriptive Characteristics of the Study Population

A total of 17 508 people with CD were included (Table 1); 261 (1.49%) Chagas-specific deaths were recorded during follow-up. Hospitalization occurred in 3.72% (652/17 508) of all cases, with 11.66% (76/652 cases) dying during hospitalization (χ^2^ test *P* = .001). The median LOS was 6 days (IQR, 3–14.25), increasing to 15 days (IQR, 6–28) among those who died from CD and 10 days (IQR, 5–19.25) among those who died from other causes (Mann-Whitney *U* test *P* < .001). Men represented 37.79% (6617/17 508) of the cohort, with a crude chagas-specific mortality proportion of 2.16% (143/6617; χ^2^ test *P* < .001; 8.80% other deaths). Chagas-specific mortality increased progressively with age, from no deaths among individuals younger than 35 years to 3.1% in those aged 65–74 years and 5.5% among those aged ≥75 years. In the ≥75-year group, 95 of 1717 individuals died from CD, while 355 died from other causes. Chronic disease was the predominant clinical stage (91.22%; 15 971/17 508), and among ICD-10 diagnoses, Z22.8 (predominantly asymptomatic or screening-detected infections) was the most frequent classification (59.33%; 10 351/17 446 cases). The highest crude Chagas-specific mortality proportion by ICD-10 category was observed in B57.3 (chronic digestive form) (4.79%; 26/543 cases).

**Table 1.** Distribution of Sociodemographic, Clinical, and Hospitalization-related Variables Among Individuals Diagnosed With Chagas Disease in Chile (2007–2021), According to Mortality Status

Covariates, Total	Total	Chagas-specific Death	Other-death	Alive	*P* Value^c,e^
	n (%)^a^	n (%)^a^	n (%)^a^	n (%)^a^	
Overall	17 508 (100)	261(1.49)	1021(5.83)	16 226(92.68)	…
Hospitalization					<.001
Hospitalized	652 (3.72)	76 (11.66)	74 (11.35)	502 (76.99)	…
Not hospitalized	16 856 (96.28)	185 (1.10)	947 (5.62)	15 724 (93.28)	…
LOS (days)^d^, n = 652	6.0 (3.0–14.25)	15.0 (6.0–28.0)	10.0 (5.0–19.25)	5.0 (2.0–11.75)	<.001
Sex					<.001
Men	6619 (37.79)	143 (2.16)	582 (8.80)	5894 (89.04)	…
Women	10 889 (62.19)	118 (1.08)	439 (4.03)	10 332 (94.88)	…
Age					<.001
<15	368 (2.1)	0 (0.0)	3 (1.1)	365 (98.9)	…
15–24	1082 (6.2)	0 (0.0)	4 (2.4)	1078 (97.6)	…
25–34	2427 (14.0)	0 (0.0)	11 (2.9)	2416 (97.1)	…
35–44	2656 (15.3)	2 (0.1)	29 (4.1)	2625 (95.8)	…
45–54	3389 (19.5)	19 (0.5)	75 (5.1)	3295 (94.4)	…
55–64	3330 (19.1)	46 (1.4)	167 (7.9)	3117 (90.7)	…
65–74	2462 (14.0)	81 (3.1)	290 (11.9)	2091 (85.0)	…
≥75	1794 (9.9)	113 (5.5)	442 (20.7)	1239 (73.8)	…
Diagnostic stage					.016
Acute	1537 (8.78)	26 (1.69)	114 (7.42)	1397 (90.89)	…
Chronic	15 971 (91.22)	235 (1.47)	907 (5.68)	14 829 (92.85)	…
Diagnoses^b^, n = 17 446					<.001
B57.0	134 (0.77)	2 (1.49)	6 (4.48)	126 (94.03)	…
B57.1	1399 (8.02)	24 (1.72)	108 (7.72)	1267 (90.56)	…
B57.2	3047 (17.47)	85 (2.79)	308 (10.11)	2654 (87.10)	…
B57.3	543 (3.11)	26 (4.79)	57 (10.50)	460 (84.71)	…
B57.4	15 (0.09)	0 (0.00)	1 (6.67)	14 (93.33)	…
B57.5	1869 (10.71)	33 (1.77)	124 (6.63)	1712 (91.60)	…
P00.2	88 (0.50)	0 (0.00)	0 (0.00)	88 (100.0)	…
Z22.8	10 351 (59.33)	91 (0.88)	417 (4.03)	9843 (95.09)	…
Zone					<.001
Northern macrozone	6138 (35.06)	83 (1.35)	327 (5.33)	5728 (93.32)	…
Central macrozone	8642 (49.36)	149 (1.72)	582 (6.73)	7911 (91.54)	…
Southern macrozone	107 (0.61)	1 (0.93)	2 (1.87)	104 (97.20)	…
Metropolitan region	2621 (14.97)	28 (1.07)	110 (4.20)	2483 (94.73)	…

^a^Values are presented as absolute numbers and percentages (%).

^b^ICD-10 codes were used to classify Chagas disease diagnoses: Z22.8, predominantly asymptomatic or screening-detected infections; B57.2, chronic cardiac form; B57.3, chronic digestive form; B57.4, chronic disease affecting nervous system; B57.5, other organ involvement; B57.0 and B57.1, acute forms with and without cardiac involvement, respectively; P00.2, congenital.

^c^Categorical variables were compared using the χ^2^ test. When expected frequencies were low, global associations (eg, between clinical diagnosis or geographic region and vital status) were evaluated using Fisher´s exact test with a Monte Carlo simulation (10 000 replicates).

^d^LOS: Length of stay, presented as median and interquartile range (IQR). The Kruskal-Wallis test assessed differences in LOS between deceased and surviving patients.

^e^A *P* value <.05 was considered statistically significant.

### Notified Incidence Over Time and Demographic Differences

**Figure 1.**
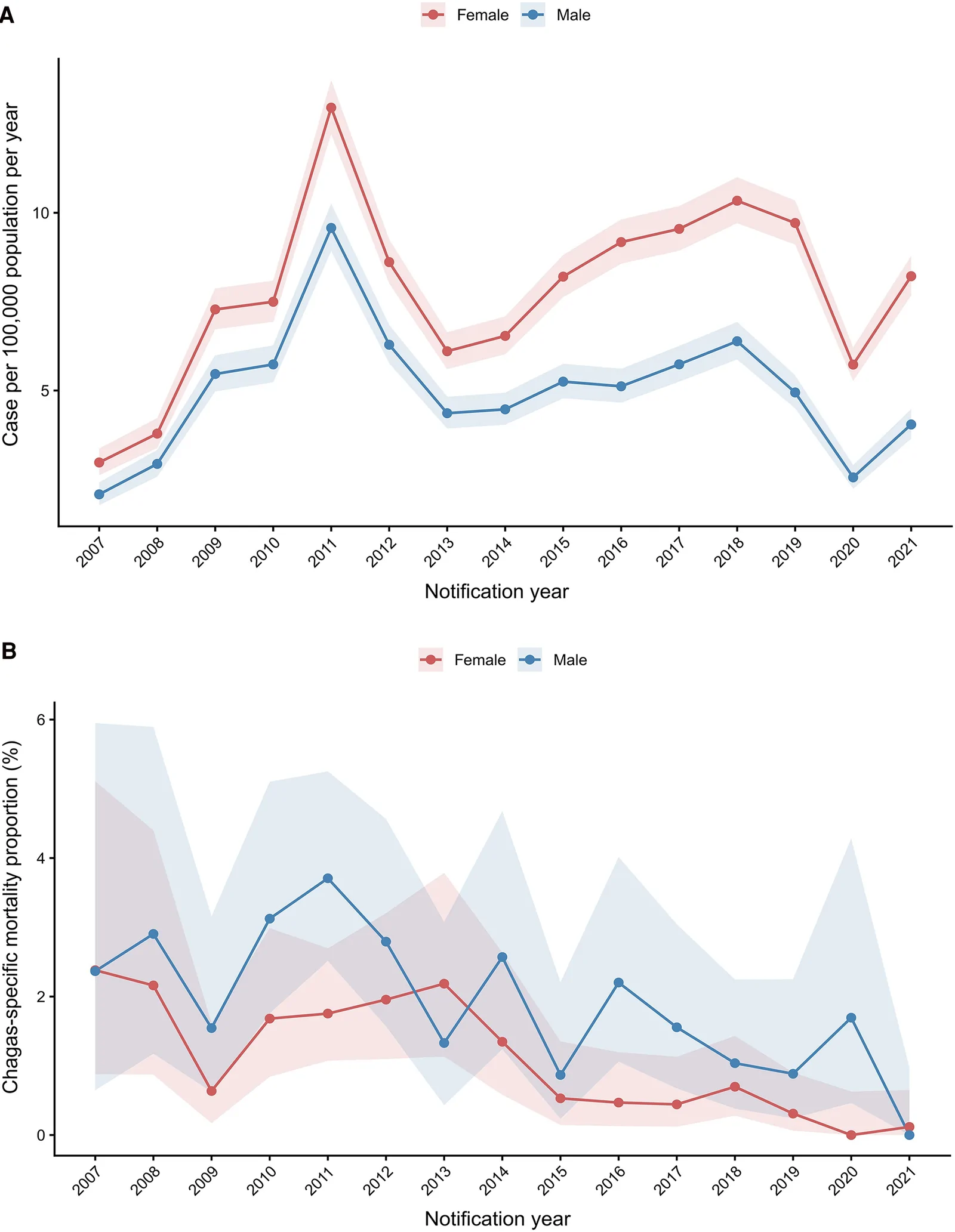
Notified incidence (*A*) and crude Chagas-specific mortality proportion (%) (*B*) of Chagas disease in Chile from 2007 to 2021, stratified by sex. Notes: ^a^ (*A*) Annual incidence rate of Chagas disease stratified by sex (male vs female), with bars representing yearly estimates and vertical lines indicating 95% CIs. ^b^ (*B*) Chagas-specific death proportion by sex for the same study period, displayed as annual estimates with corresponding 95% CI bars.

The average annual notified incidence rate from 2007 to 2021 was 6.45 per 100 000 population per year (95% CI 5.93–6.96), remaining consistently higher among women than men (Figure 1). Incidence increased from 2007 to a peak in 2011, then stabilized between 2014 and 2019 (ranging from 5.12 to 10.34 per 100 000 population per year) (Supplementary Table 1). Over the 2007–2021 period, 51.5% of all reported cases occurred between March and August (austral autumn-winter).

Age-stratified notified incidence was highest among adults aged 45–54 and 55–64 years, peaking in 2011 at 2.80 (95% CI 2.55–3.06) and 2.53 (95% CI 2.29–2.77) cases per 100 000 population per year, respectively. Adults aged 25–34 years also showed increasing notified incidence in later years, reaching 1.45 (95% CI 1.28–1.62) per 100 000 population in 2021. In contrast, individuals aged <15 and 15–24 years consistently showed the lowest notified incidence throughout the study period (see Supplementary Table 2 and Supplementary Figure 2).

Chronic-stage diagnoses consistently dominated the epidemiological profile, remaining high and stable over time. After 2012, the number of acute cases remained below 0.2 per 100 000 population per year (Supplementary Table 3 and Supplementary Figure 3). Z22.8 remained the predominant diagnostic category throughout the study period (Supplementary Table 4 and Supplementary Figure 4). The Central macrozone showed the sharpest spike, peaking at 7.80 (95% CI 7.39–8.23) per 100 000 population in 2011, while the Northern macrozone peaked later at 3.78 (95% CI 3.50–4.07) per 100 000 population in 2018 (Supplementary Table 5 and Supplementary Figure 5).

### Crude Chagas-specific Death Patterns

The overall Chagas-specific death proportion was 1.49% (n = 261). The death proportions were higher among men, with a peak of 3.77% (95% CI 2.66–5.30) in 2011 (see Supplementary Table 1, Figure 1, and Supplementary Figure 1), and increased sharply with age, with the highest annual Chagas-specific mortality proportions observed among individuals aged ≥75 years in 2008 (16.13%; 95% CI 5.45–33.73). Elevated Chagas-specific mortality proportions were also observed in the oldest age groups, particularly among those aged 65–74 and ≥75 years during the early notification years (Supplementary Table 6 and Supplementary Figure 2). The highest category-specific death proportion was observed in B57.3 (chronic digestive form) (Supplementary Table 7 and Supplementary Figure 4).

Hospitalized patients showed consistently higher Chagas-specific mortality proportions than nonhospitalized patients, with the highest estimates observed in the early notification years: 21.43% in 2007 (95% CI 4.66–50.80) and 22.22% in 2008 (95% CI 6.41–47.64). During 2010–2016, estimates generally remained above 10%, except in 2015 (5.71%) (Supplementary Table 8 and Supplementary Figure 6-7). Among hospitalized notified cases, Chagas-specific and all-cause mortality proportions were generally higher among men and older adults, particularly those aged 65–74 and ≥75 years (Supplementary Figure 8-9). Mortality proportions were also more consistently elevated among patients diagnosed in the chronic stage, whereas estimates for acute disease were unstable because of small annual denominators (Supplementary Tables 9–12 and Supplementary Figure 10-11).

### Competing-risks Analysis: Cumulative Incidence and Fine–Gray Regression

**Figure 2.**
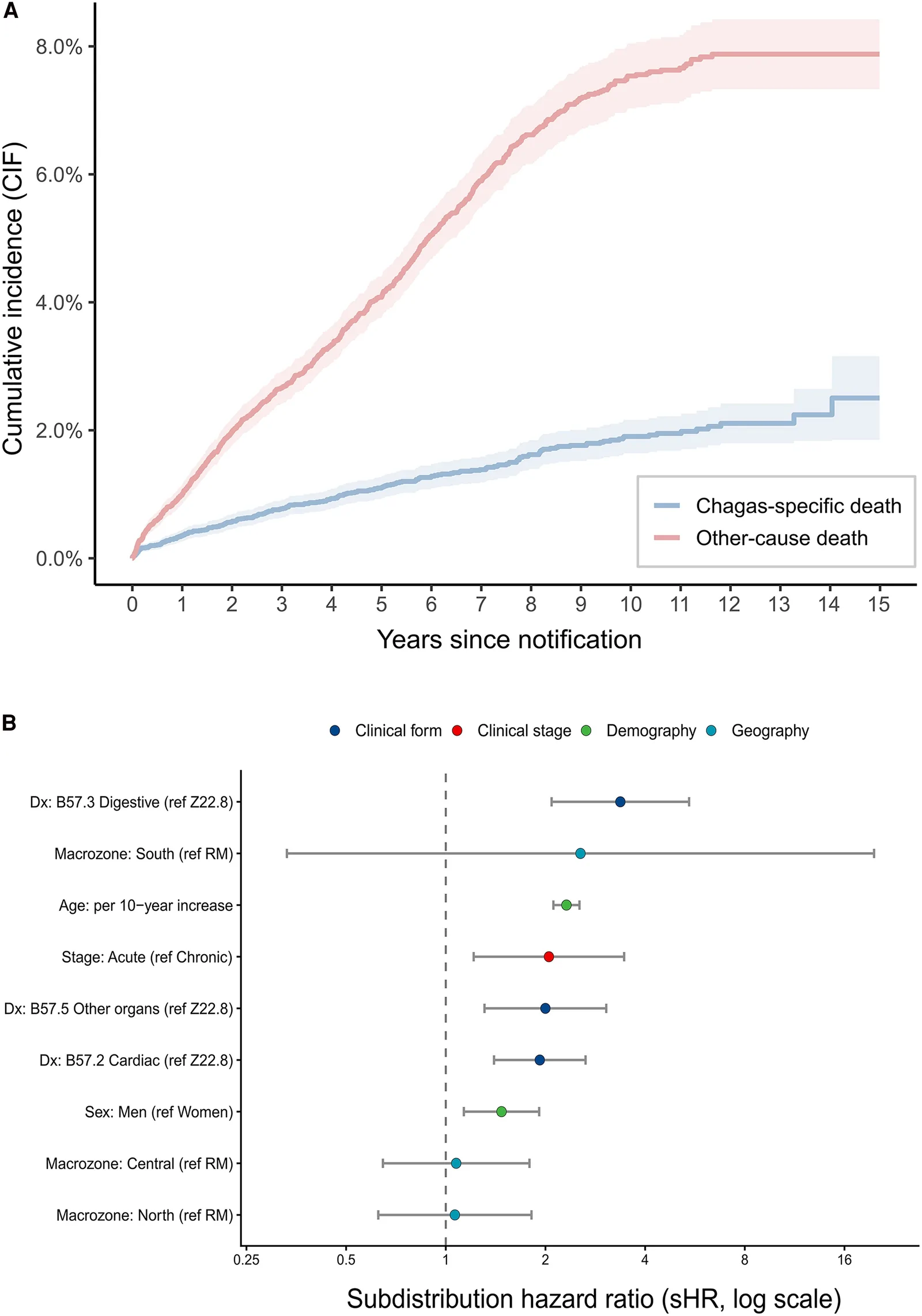
Competing-risks analysis of Chagas disease in Chile from 2007 to 2021: cumulative incidence and Fine–Gray regression. Notes: ^a^(*A*) Cumulative incidence functions (CIF) for Chagas-specific death and other-cause death from notification, accounting for competing risks. ^b^(*B*) Adjusted subdistribution hazard ratios (SHR) for Chagas-specific death estimated using the multivariable Fine–Gray model (with statistical reference categories as labeled). ^c^CIF is expressed as a percentage over follow-up; time is shown in years since notification. ^d^CIs are 95%; categories are color-coded by domain (clinical form, clinical stage, demography, geography).

Chagas-specific mortality concentrated in distinct demographic and clinical strata and showed a sustained accumulation over follow-up, with most of the increase occurring in the first decade (ΔCIF 0–5 years 1.11%; 5–10 years 0.80%; 10–15 years 0.60%) (Figure 2). Ten-year Chagas-specific cumulative incidence increased sharply with age, reaching 4.80% among individuals aged 65–74 years and 8.89% among those aged ≥75 years (Gray *P* < .005) (see Supplementary Table 13 and Supplementary Figure 13) and was higher in men (2.66%; vs women 1.41%; Gray *P* < .005) (Supplementary Figure 14). By diagnostic stage, 10-year Chagas-specific CIF did not differ between acute and chronic notifications (acute 1.70%; Gray *P* = .432) (Supplementary Figure 15). Marked differences were also observed by ICD-10 category, peaking in the chronic digestive form (B57.3) (6.49%) and remaining elevated in chronic cardiac disease (B57.2) (2.93%) (among predominantly asymptomatic or screening-detected infections classified as Z22.8, 1.47%: Gray *P* < .005) (Supplementary Figure 16). B57.3 comprised a relatively small subgroup (n = 543) and should be interpreted as an administrative diagnostic category rather than a clinically validated phenotype.

Geographic variation in Chagas-specific death was modest (Metropolitan region 1.06% vs macrozones 2.06–2,61%; Gray *P* = .138) (Supplementary Figure 17). Other-cause mortality was higher overall and showed clearer stratification, particularly by age, increasing from low levels in younger groups to 15.88% among individuals aged 65–74 years and 37.23% among those aged ≥75 years at 10 years (Gray *P* < .005) and by sex, with higher 10-year other-cause CIF in men than in women (10.53% vs 5.58%; Gray *P* < .005).

In univariable Fine–Gray models (Table 2), Chagas-specific mortality risk was strongly associated with age, with each 10-year increase in age associated with a higher subdistribution hazard (sHR 2.32; 95% CI 2.14–2.52, *P* < .005), male sex (Men: sHR 1.89; 95% CI 1.46–2.45, *P* < .005), and chronic clinical forms, particularly cardiac and digestive involvement (B57.2: sHR 2.55; 95% CI 1.85–3.54, *P* < .005; and B57.3: sHR 5.90; 95% CI 3.67–9.52, *P* < .005). Chronic CD with other organ involvement (B57.5) showed weaker evidence (sHR 1.48; 95% CI .97–2.2, *P* = .065), while acute CD without cardiac involvement (B57.1) was not clearly different from the reference category (sHR 1.39; 95% CI .87–2.23, *P* = .160). By geography, the Central macrozone showed a higher subdistribution risk (sHR 1.73; 95% CI 1.11–2.70, *P* = .015), whereas North and South were not clearly different (North: sHR 1.47; 95% CI .92–2.36, *P* = .110; and South: sHR 1.43; 95% CI .20–10.48, *P* = .720).

**Table 2.** Univariable and Multivariable Fine–Gray Models Among Patients Diagnosed With Chagas Disease in Chile (2007–2021)

Covariates	10-y CIF (Chagas-specific Death)^b^, %	Univariable Fine–Gray Regression			Multivariable Fine–Gray Regression		
		sHR^c^	95% CI	*P* Value^d^	sHR^c^	95% CI	*P* Value^d^
Age							
Age categories	See supplementary table N° 13	…	…	…	…	…	…
Age, per 10-y increase	-	2.32	(2.14–2.52)	<.005	2.31	(2.11–2.53)	<.005
Sex							
Men (vs.. Women)^e^	2.66	1.89	(1.46–2.45)	<.005	1.47	(1.13–1.91)	<.005
Diagnoses^a^							
B57.1 (vs.. Z22.8)^e^	1.70	1.39	(0.87–2.23)	.160	…	…	…
B57.2	2.93	2.55	(1.85–3.54)	<.005	1.92	(1.39–2.64)	<.005
B57.3	6.49	5.90	(3.67–9.52)	<.005	3.36	(2.08–5.43)	<.005
B57.5	1.88	1.48	(0.98–2.27)	.065	1.99	(1.30–3.05)	<.005
Diagnostic stage							
Acute stage (vs.. Chronic stage)^e^	1.70	0.86	(0.56–1.33)	.500	2.04	(1.21–3.45)	.007
Zones							
Central macrozone (vs.. MR)^e^	2.06	1.73	(1.11–2.70)	.015	1.07	(0.64–1.78)	.780
South macrozone	2.61	1.43	(0.20–10.48)	.720	2.55	(0.33–19.65)	.370
Northern macrozone	2.08	1.47	(0.92–2.36)	.110	1.06	(0.62–1.81)	.820

^a^ICD-10 codes were used to classify Chagas disease diagnoses: Z22.8, predominantly asymptomatic or screening-detected infections; B57.2, chronic cardiac form; B57.3, chronic digestive form; B57.4, chronic disease affecting nervous system; B57.5, other organ involvement; B57.0 and B57.1, acute forms with and without cardiac involvement, respectively; P00.2, congenital.

^b^Cumulative incidence function (CIF) at 10 y is expressed as a percentage.

^c^Subdistribution hazards ratios correspond to univariable and multivariable competing-risks regression models for Chagas-specific death.

^d^Statistical significance was defined as *P* < .05.

^e^vs.: reference category.

After mutual adjustment, the multivariable analysis (Table 2, Supplementary Table 14, and Figure 2) showed that age remained strongly associated with Chagas-specific mortality (per 10-year increase: sHR 2.31; 95% CI 2.11–2.53, *P* < .005), and male sex (Men: sHR 1.47; 95% CI 1.13–1.91, *P* < .005) remained independently associated with higher Chagas-specific mortality. The associations for cardiac and digestive forms persisted but attenuated (B57.2: sHR 1.92; 95% CI 1.39–2.64, *P* < .005; and B57.3: sHR 3.36; 95% CI 2.08–5.43, *P* < .005), and chronic CD with other organ involvement (B57.5) became clearly associated (sHR 1.99; 95% CI 1.30–3.05, *P* < .005). The acute stage showed an increased adjusted subdistribution risk (sHR 2.04; 95% CI 1.21–3.45, *P* = .007). By macrozone, the univariable Central association was not retained after adjustment (sHR 1.07; 95% CI .64–1.78, *P* = .780), and neither North nor South showed clear adjusted differences (North: sHR 1.06; 95% CI .62–1.81; *P* = .820; and South: sHR 2.55; 95% CI .33–19.65, *P* = .370).

### Cause-specific Cox Regression

In univariable cause-specific Cox models (Table 3), age showed a strong gradient with Chagas-specific mortality. Each 10-year increase in age was associated with a higher instantaneous hazard of Chagas-specific death among individuals still at-risk (HR 2.53; 95% CI 2.31–2.78, *P* < .005). Higher hazards were also observed among men (HR 1.93; 95% CI 1.21–3.06, *P* < .005) and in organ-involving clinical forms compared with predominantly asymptomatic or screening-detected infections classified as Z22.8 (Chronic cardiac form (B57.2): HR 2.57; 95% CI 1.89–3.48, *P* < .005; and chronic digestive form (B57.3): HR 6.09; 95% CI 3.15–11.76, *P* < .005). Hospitalization showed a strong dose-response gradient (any hospitalization: HR 10.45; 95% CI 6.69–16.31; *P* < .005, 1 hospitalization: HR 8.18; 95% CI 4.89–13.68, *P* < .005; 2 hospitalizations: HR 18.20; 95% CI 10.68–31.03, *P* < .005; and 3+ hospitalizations: HR 34.30; 95% CI 21.15–55.62, *P* < .005), while macrozones were not clearly associated.

**Table 3.** Univariable and Multivariable Cause-specific Cox Models for Chagas-specific Mortality Among Patients Diagnosed With Chagas Disease in Chile (2007–2021)

Covariates	Univariable Cause-specific Cox Regression^c^			Multivariable Cause-specific Cox Regression^d^		
	HR^b^	95% CI	*P* Value	HR^b^	95% CI	*P* Value^e^
**Age**						
Age, per 10-y increase	2.53	(2.31–2.78)	<.005	2.43	(2.21–2.68)	<.005
**Sex**						
Men (vs.. Women)^f^	1.93	(1.21–3.06)	.005	1.30	(0.93–1.81)	.128
**Diagnoses^a^**						
B57.2 (vs.. Z22.8)^f^	2.57	(1.89–3.48)	<.005	1.77	(1.44–2.19)	<.005
B57.3	6.09	(3.15–11.76)	<.005	2.45	(1.82–3.31)	<.005
B57.5	1.46	(0.78–2.74)	.235	1.90	(1.11–3.25)	.019
**Hospitalization**						
Any hospitalization	10.45	(6.69–16.31)	<.005	…	…	…
1 hospitalization	8.18	(4.89–13.68)	<.005	9.36	(5.63–15.5)	<.005
2 hospitalizations	18.20	(10.68–31.03)	<.005	19.28	(10.3–35.9)	<.005
3+ hospitalizations	34.30	(21.15–55.62)	<.005	83.94	(40.9–172.2)	<.005
**Zones**						
Central macrozone (vs.. MR)^f^	1.75	(1.46–2.10)	<.005	0.85	(0.69–1.04)	.123
Northern macrozone	1.49	(1.05–2.10)	.023	0.88	(0.63–1.21)	.447
South macrozone	1.42	(0.22–9.26)	.707	1.95	(0.28–13.2)	.494

^a^ICD-10 codes were used to classify Chagas disease diagnoses: Z22.8, predominantly asymptomatic or screening-detected infections; B57.2, chronic cardiac form; B57.3, chronic digestive form; B57.4, chronic disease affecting nervous system; B57.5, other organ involvement; B57.0 and B57.1, acute forms with and without cardiac involvement, respectively; P00.2, congenital.

^b^Hazard ratios (HRs) are cause-specific hazards from Cox proportional hazard models for Chagas-specific death.

^c^Univariable models were fitted separately for each covariate.

^d^The multivariable model included baseline covariates and modeled hospitalization as a time-varying prognostic marker using a start-stop structure (AIC 3319.73).

^e^Statistical significance was defined as *P* < .05.

^f^vs.: reference category.

**Figure 3.**
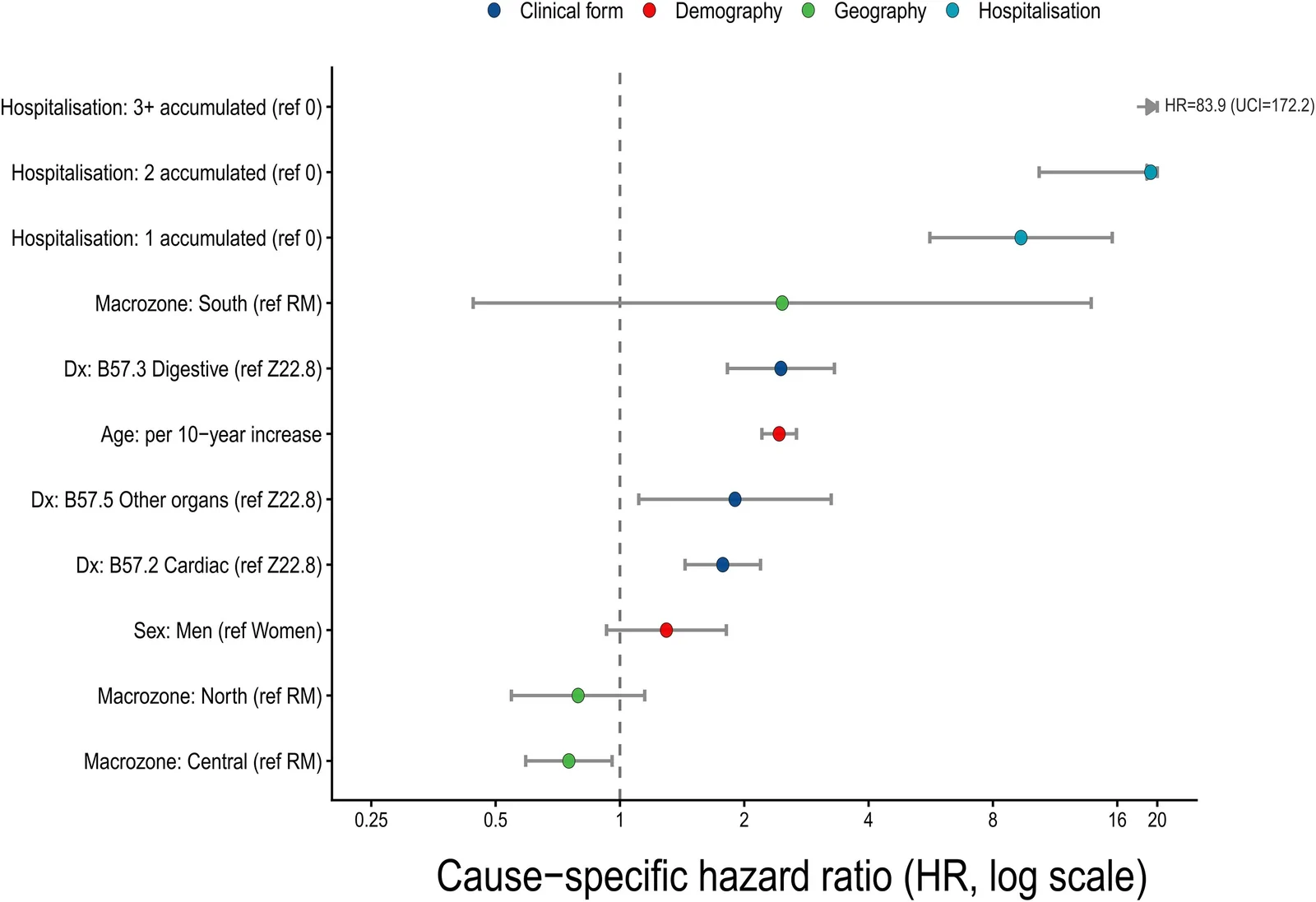
Adjusted cause-specific Cox regression model for Chagas-specific mortality, including recurrent hospitalization as a time-varying prognostic marker. Notes: ^a^ Adjusted cause-specific hazard ratios (HRs) for Chagas-specific death were estimated using a multivariable cause-specific Cox model stratified by diagnostic stage, with other-cause deaths censored at the time of occurrence and reference categories as labeled. ^b^ Hospitalization was modeled as a time-varying accumulated number of admissions, with no previous hospitalization as the reference category. Estimates should be interpreted as prognostic associations rather than causal effects. ^c^ Hazard ratios are presented on a logarithmic scale with 95% CIs. CIs extending beyond the plotting range were truncated for visualization, as indicated by arrows. ^d^ Categories are color-coded by domain: clinical form, demography, geography, and hospitalization.

After multivariable adjustments (Table 3, Figure 3, and Supplementary Table 15), Chagas-specific mortality increased steeply with age among individuals who remained under risk (per 10-year increase: HR 2.43; 95% CI 2.21–2.68, *P* < .005) and diagnostic categories retained a marked gradient (Chronic cardiac form [B57.2]: HR 1.77; 95% CI 1.44–2.19, *P* < .005; Chronic digestive form [B57.3]: 2.45; 95% CI 1.82–3.31; *P* < .005; and chronic CD with other organ involvement [B57.5]: HR 1.90; 95% CI 1.11–3.25, *P* = .019). The sex association attenuated after adjustment (men: HR 1.30; 95% CI .93–1.81, *P* = .128). In contrast, the number of hospital admissions showed a marked graded association with the cause-specific hazard of Chagas-specific death (1 hospitalization: HR 9.36; 95% CI 5.63–15.55, *P* < .005; 2 hospitalizations: HR 19.28; 95% CI 10.35–35.91, *P* < .005; 3 or more hospitalizations: HR 83.94; 95% CI 40.91–172.23, *P* < .005). These estimates indicate that recurrent hospitalization may serve as a prognostic marker of advanced clinical severity rather than an independent causal risk factor. Macrozone estimates remained imprecise and were not independently associated with Chagas-specific mortality.

Cox diagnostics indicated improved model fit, with no global violation of proportional hazards (Supplementary Table 16).

## DISCUSSION

To our knowledge, this is the most comprehensive nationwide cohort study of CD mortality conducted in Chile. Although Chagas-specific death was infrequent in absolute terms, its cumulative incidence increased with age and organ involvement and was higher among men. Chronic digestive and cardiac categories showed the highest cumulative incidence, whereas recurrent hospitalization was strongly associated with the cause-specific hazard of death. These findings delineate a risk-stratified mortality profile and identify clinically recognizable groups that may benefit from intensified assessment and follow-up.

Most patients were in the chronic phase, consistent with long-term data from Latin America [7], and the older age distribution reflected Chile´s aging seropositive population [18]. Women represented 62.2% of the cohort, and 59.3% of cases were classified as Z22.8, reflecting the important contribution of antenatal, blood-donor, and other screening programs to case detection [19, 20]. Chronic cardiac disease (B57.2) accounted for 17.5% of cases, while chronic CD with other organ involvement (B57.5) represented 10.7%. However, these administrative proportions should not be interpreted as estimates of the underlying prevalence of organ involvement, as they may reflect notification practices, the predominance of screening-detected infections, and differences in diagnostic assessment [14]. The concentration of notified cases in the Central macrozone may similarly reflect historical migration and regional differences in access to diagnostic services [13].

Notified incidence peaked in 2011 and subsequently stabilized between 5.0 and 10.4 cases per 100 000 population per year, remaining higher among women. The increasing proportion of cases classified as predominantly asymptomatic or screening-detected probably reflects expanded surveillance following the certification of interruption of domiciliary vectorial transmission, rather than a resurgence of vector-borne infection [13, 21, 22]. Regional differences may also reflect historical endemicity, migration, and unequal access to screening and diagnosis [21]. These patterns support Chile´s transition from vector control toward targeted screening and chronic disease management, particularly for pregnant women, blood donors, migrants, and other groups at risk [6, 23–25].

The overall crude Chagas-specific death proportion was 1.49% (261/17 508). Higher proportions among men, older individuals, hospitalized patients, and those classified with cardiac or digestive involvement were broadly consistent with findings from Brazil, Spain, and Colombia [26–35]. Individuals classified with chronic Chagas heart disease (B57.2), used as an administrative proxy for chronic Chagas cardiomyopathy, had a higher crude death proportion than those in the predominantly asymptomatic or screening-detected Z22.8 category. This finding is consistent with the well-established poor prognosis associated with chronic Chagas cardiomyopathy [30–34]. Nevertheless, these estimates were unadjusted proportions and should not be interpreted as cumulative risks. The apparent decline after 2014 probably reflects changes in case ascertainment and available follow-up. In particular, the absence of Chagas-specific deaths among cases notified in 2021 is likely to reflect their short follow-up. Pandemic-related disruption may also have influenced diagnosis, notification, healthcare use, and outcome ascertainment during the final study years [26, 27, 36].

Competing-risks analyses showed that Chagas-specific cumulative incidence increased with age and was higher among men, consistent with previous studies reporting poorer outcomes among older patients and men with CD [13, 27, 28, 37]. Chronic digestive disease (B57.3) showed the highest cumulative incidence and adjusted subdistribution hazard, followed by chronic cardiac disease (B57.2). The B57.3 finding should be interpreted cautiously: this category comprised only 543 individuals and 26 Chagas-specific deaths, and may preferentially identify patients with clinically manifest or advanced digestive disease. Concomitant cardiac or other organ involvement may also have been incompletely captured in the notification record. The estimate, therefore, represents an association within a relatively small and administratively selected subgroup, rather than evidence that digestive disease is the principal driver of Chagas-specific mortality. Chronic cardiac disease was substantially more prevalent and remains the most clinically established determinant of poor prognosis, particularly among older patients and those with advanced disease [13, 27, 28, 38–40].

Cause-specific Cox models showed a graded association between recurrent hospitalization and Chagas-specific death. This association should not be interpreted causally, as repeated admission probably identifies patients with advanced organ involvement, clinical decompensation, comorbidities, or greater healthcare needs. Reverse causation is also possible because patients experiencing clinical deterioration or approaching death are more likely to require repeated hospital care. As information on disease severity, cardiac function, comorbidities, and reasons for admission was unavailable, we could not determine how much of this association was explained by these factors. This interpretation is consistent with studies from Brazil and Chile linking recurrent admissions and greater clinical complexity to higher fatality [13, 27, 36]. Nevertheless, recurrent hospitalization may provide a pragmatic prognostic marker for identifying patients who require intensified assessment and structured postdischarge follow-up. Considered jointly with age, sex, and recorded clinical phenotype, it may support a risk-stratified approach to chronic CD care [18, 37, 41, 42].

This study has limitations. It relied on administrative and diagnostic registry data and may therefore underrepresent mild or asymptomatic infections and misclassify clinical presentation or cause of death, potentially leading to under-ascertainment of Chagas-specific mortality. Although all cases met national laboratory confirmation criteria, patient-level information on diagnostic assays and confirmatory algorithms was unavailable. Temporal and regional differences in screening coverage, laboratory capacity, diagnostic access, and the management of discordant results may have affected case ascertainment. Moreover, ICD-10 categories recorded at notification were not independently validated against medical records and may not have captured concomitant organ involvement.

Surveillance, screening coverage, healthcare access, and clinical management also changed during the study period, and the COVID-19 pandemic may have further disrupted diagnosis and case notification. We could not distinguish temporal changes in the underlying epidemiology from changes in detection, coding, and healthcare delivery. The linked databases additionally lacked information on cardiac function, comorbidities, antiparasitic treatment, socioeconomic circumstances, and continuity of care. Residual confounding is therefore likely, particularly for the associations with the ICD-10 category and recurrent hospitalization. Adjusted sHRs and cause-specific HRs should consequently be interpreted as associations conditional on measured covariates rather than causal effects or fully independent prognostic effects. Nevertheless, linkage across national databases provides a valuable overview of the evolving clinical and demographic profile of CD in Chile.

Chile illustrates the post-transmission challenge facing several Latin American countries: despite declining vectorial transmission, an aging population with chronic infection remains at risk of potentially preventable mortality. Our findings support integrating CD into chronic-care strategies, with intensified assessment and structured follow-up for patients with organ involvement or recurrent hospitalization. This approach is consistent with the Sustainable Development Goals and the WHO 2021–2030 NTD roadmap, which emphasize morbidity management and stronger health systems beyond transmission interruption [16].

## Supplementary Material

ofag502_Supplementary_Data

## Supplementary Data

Supplementary materials are available at *Open Forum Infectious Diseases* online. Consisting of data provided by the authors to benefit the reader, the posted materials are not copyedited and are the sole responsibility of the authors, so questions or comments should be addressed to the corresponding author.
