# Supplementary material for: Chagas-specific Death and Competing Risk of Mortality in Chile: A Nationwide Retrospective Cohort Study, 2007–2021
Source: Open Forum Infect Dis. 2026 Aug 14;13(8):ofag502. doi: 10.1093/ofid/ofag502 (PMC13492210; doi:10.1093/ofid/ofag502)
Supplement: Supplementary file 1 [file ofag502_supplementary_data.zip › Supplementary_material_manucrispt_July18.pdf]

Supplementary material

Chagas-specific death and competing risk of mortality in Chile: A nationwide retrospective cohort study, 2007-2021

Franco Fernández-Guardiola, Claudia T. Codeço, Natalia Vergara, Jorge Vilches, Diego Sandoval-Vargas, Eduardo A. Undurraga, Mauricio Canals, Kasim Allel

Content

Section 1. Abbreviations used in the Supplementary material. 3

Section 2. Study flow diagram 4

Section 3. Supplementary text 5

Supplementary text 1. Laboratory methods for Chagas disease diagnosis in Chile.. 5

Section 4. Supplementary tables 6

Supplementary Table 1. Yearly incidence and Chagas-specific mortality proportion by sex among Chagas disease cases in Chile, 2007–2021. 6

Supplementary Table 2. Yearly notified incidence by age groups among Chagas disease cases in Chile, 2007–2021 7

Supplementary Table 3. Yearly incidence and Chagas-specific mortality proportion by diagnostic stage among Chagas disease cases in Chile, 2007–2021 10

Supplementary Table 4. Yearly notified incidence by ICD-10 codes among Chagas disease cases in Chile, 2007–2021 11

Supplementary Table 5. Yearly notified incidence by geographical zone among Chagas disease cases in Chile, 2007–2021 14

Supplementary Table 6. Yearly Chagas-specific mortality proportion by age groups among Chagas disease cases in Chile, 2007–2021 16

Supplementary Table 7. Yearly Chagas-specific mortality proportion (%) by ICD-10 codes among Chagas disease cases in Chile, 2007–2021..................................... 24

Supplementary Table 8. Yearly Chagas-specific mortality proportion by hospitalisation status among Chagas disease cases in Chile, 2007–2021 26

Supplementary Table 9. Yearly Chagas-specific mortality proportion (%) by age groups among hospitalised patients with Chagas disease cases in Chile, 2007–2021 27

Supplementary Table 10. Yearly Chagas-specific mortality proportion (%) by sex among hospitalised patients with Chagas disease cases in Chile, 2007–2021......... 30

Supplementary Table 11. Yearly Chagas-specific mortality proportion (%) by diagnostic stage among hospitalised patients with Chagas disease cases in Chile, 2007–2021 31

Supplementary Table 12. Yearly Chagas-specific mortality proportion by ICD-10 codes among hospitalised patients with Chagas disease cases in Chile, 2007–2021 32

Supplementary Table 13. Five- and ten-year cumulative incidence of Chagas-specific and other-cause death by age group. 34

Supplementary Table 14. Sequential multivariable Fine-Gray subdistribution hazards models for Chagas-specific mortality in notified patients with Chagas disease, Chile 2007–2021. 34

Supplementary Table 15. Multivariable cause-specific Cox regression models for Chagas-specific mortality in notified patients with Chagas disease, Chile 2007–2021 36

Supplementary Table 16. Cox model assumption testing. 38

Section 5. Supplementary figures 39

Supplementary Figure 1. Annual Chagas-specific and all-cause mortality proportion (%) by sex and notification year, Chile, 2007–2021. 39

Supplementary Figure 2. Notified incidence and Annual Chagas-specific and all-cause mortality proportion (%) of Chagas disease in Chile from 2007 to 2021, stratified by age group. 40

Supplementary Figure 3. Notified incidence and Annual Chagas-specific and all-cause mortality proportion (%) of Chagas disease in Chile from 2007 to 2021, stratified by diagnostic stage. 41

Supplementary Figure 4. Notified incidence and Annual Chagas-specific and all-cause mortality proportion (%) of Chagas disease in Chile from 2007 to 2021, stratified by ICD-10 category. 42

Supplementary Figure 5. Notified incidence and Annual Chagas-specific and all-cause mortality proportion (%) of Chagas disease in Chile from 2007 to 2021, stratified by geographic zones 43

Supplementary Figure 6. Annual Chagas-specific and all-cause mortality proportion (%) by hospitalisation status and notification year, Chile, 2007–2021 44

Supplementary Figure 7. Annual Chagas-specific and all-cause mortality proportion (%) by number of hospitalisations and notification year, Chile, 2007–2021. 45

Supplementary Figure 8. Annual Chagas-specific and all-cause mortality proportion (%) among hospitalised patients by sex and notification year, Chile, 2007–2021. 46

Supplementary Figure 9. Annual Chagas-specific and all-cause mortality proportion (%) among hospitalised patients by age and notification year, Chile, 2007–2021. 47

Section 1. Abbreviations used in the Supplementary material.

AIC = Akaike Information Criterion

CCC = Chronic Chagasic Cardiomyopathy

CD = Chagas disease

CI = Confidence interval

DALY = Disability-Adjusted Life Year

DEIS = Department of Statistics and Health Information, Ministry of Health

HR = Hazard ratio

ICD-10 = International Classification of Diseases, 10th revision.

- B57.0 = acute Chagas disease with cardiac involvement
- B57.1 = acute Chagas disease without cardiac involvement
- B57.2 = chronic Chagas disease with cardiac involvement
- B57.3 = chronic Chagas disease with digestive system involvement
- B57.4 = chronic Chagas disease with nervous system involvement
- B57.5 = chronic Chagas disease with involvement of other organs
- Z22.8 = Carrier of other infectious diseases (asymptomatic patients and used for blood donors)
- P00.2 = Foetus and newborn affected by maternal infectious and parasitic diseases.

ISP = National Institute of Health Chile

IQR = Interquartile range

LOS = Length of Stay

OS = Overall Survival

PCR = Polymerase chain reaction

Ref. = Reference category

RUN = Unique National Role (personal ID)

SARS-CoV-2 = Severe Acute Respiratory Syndrome Coronavirus 2

sHR = Subdistribution hazard ratios

WHO = World Health Organisation

Section 2. Study flow diagram

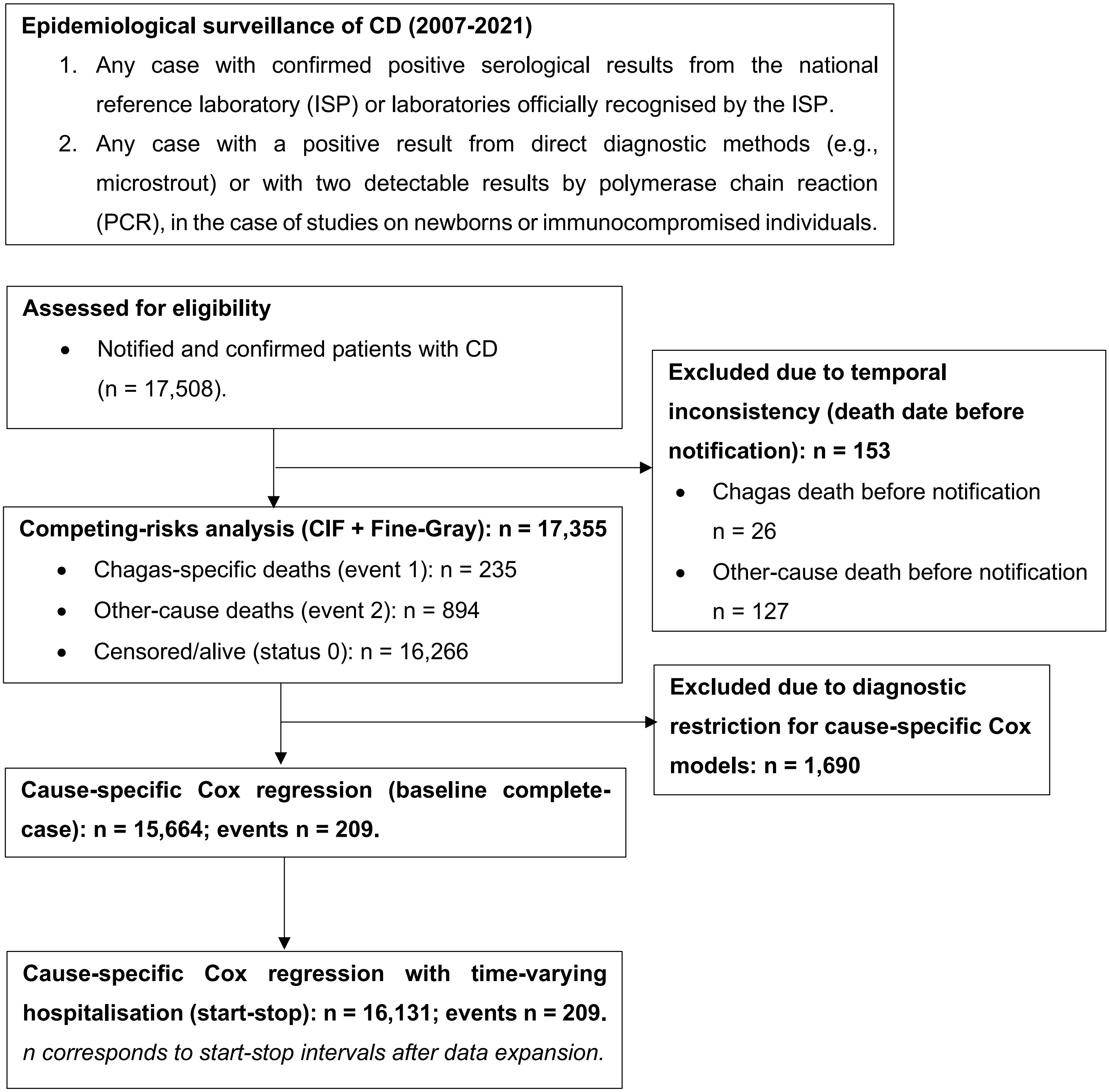

Section 3. Supplementary text

Supplementary text 1. Laboratory methods for Chagas disease diagnosis in Chile

Method	Principle	Interpretation and Recommendations
Direct Methods (Thick and thin smear, microstrout technique)	Detection of the parasite in the sample.	Results may be reported directly. If there is uncertainty, confirm with another method and send the sample to the ISP Parasitology Section. Remember that parasitaemia levels can vary by disease stage; a negative result does not rule out *T. cruzi* infection.
Molecular Methods (PCR for *T. cruzi*)	Detection of parasite genetic material in the sample.	Results can be reported directly according to patient type: Infants under 9 months and immunocompromised patients: Two positive results from different samples within one month are needed to confirm infection. Patients older than 9 months: One positive result and concordant antibody detection are required. For diagnostic uncertainty, refer samples to the ISP Parasitology Section.
Indirect Methods (Indirect hemagglutination, ELISA IgG *T. cruzi*, IFI IgG *T. cruzi*, Western Blot)	Detection of specific antibodies against the parasite.	Blood banks and diagnostic centres use ELISA IgG *T. cruzi* for initial screening, with reactive results confirmed by IFI IgG *T. cruzi*. A positive result is indicated by two out of three tests being reactive. Inconsistent results should be verified at a recognised ISP laboratory, and patients should be informed only after confirmatory testing is complete.<br><br>They correspond to the techniques for *T. cruzi* serological confirmation – surveillance.

Section 4. Supplementary tables

Supplementary Table 1. Yearly incidence and Chagas-specific mortality proportion by sex among Chagas disease cases in Chile, 2007–2021.

Year	Sex	Cases	Deceased	Population	Incidence	95%CI	Chagas-specific mortality proportion (%)	95%CI
2007	Men	172	5	8130000	2.12	(1.79-2.43)	2.91	(1.24-6.62)
2007	Women	256	6	8468000	3.02	(2.65-3.39)	2.34	(1.07-5.01)
2008	Men	243	7	8210000	2.96	(2.58-3.33)	2.88	(1.40-5.82)
2008	Women	325	7	8553000	3.80	(3.38-4.21)	2.15	(1.04-4-37)
2009	Men	457	8	8290000	5.51	(5.00-6.01)	1.75	(0.88-3.41)
2009	Women	632	4	8639000	7.32	(6.74-7.88)	0.63	(0.24-1.61)
2010	Men	484	15	8370000	5.78	(5.26-6.29)	3.10	(1.88-5.05)
2010	Women	658	12	8724000	7.54	(6.96-8.11)	1.82	(1.04-3.16)
2011	Men	822	31	8450000	9.73	(9.06-10.3)	3.77	(2.66-5.30)
2011	Women	1150	23	8798000	13.07	(12.3-13.8)	2.00	(1.33-2.98)
2012	Men	545	18	8540000	6.38	(5.84-6.91)	3.30	(2.09-5.16)
2012	Women	769	16	8905000	8.64	(8.02-9.24)	2.08	(1.28-3.35)
2013	Men	385	7	8620000	4.47	(4.02-4.91)	1.82	(0.88-3.70)
2013	Women	558	15	8992000	6.21	(5.69-6.72)	2.69	(1.63-4.38)
2014	Men	394	11	8700000	4.53	(4.08-4.97)	2.79	(1.56-4.92)
2014	Women	594	8	9087000	6.54	(6.01-7.06)	1.35	(0.68-2.63)
2015	Men	472	6	8780000	5.38	(4.89-5.86)	1.27	(0.58-2.74)
2015	Women	761	4	9191000	8.28	(7.69-8.86)	0.53	(0.20-1.34)
2016	Men	458	11	8870000	5.16	(4.69-5.63)	2.40	(1.34-4.24)
2016	Women	858	5	9297000	9.23	(8.61-9.84)	0.58	(0.24-1.35)
2017	Men	518	8	8960000	5.78	(5.28-6.27)	1.54	(0.78-3.02)
2017	Women	904	4	9459000	9.56	(8.93-10.1)	0.44	(0.17-1.13)
2018	Men	592	7	9050000	6.54	(6.01-7.06)	1.18	(0.57-2.42)
2018	Women	1011	9	9701000	10.42	(9.77-11.0)	0.89	(0.46-1.68)
2019	Men	462	4	9140000	5.05	(4.59-5.51)	0.87	(0.33-2.20)
2019	Women	973	4	9967000	9.76	(9.14-10.3)	0.41	(0.15-1.05)
2020	Men	238	5	9230000	2.58	(2.25-2.90)	2.10	(0.90-4.82)
2020	Women	589	0	10228000	5.76	(5.29-6.22)	0.00	(0.00-0.64)
2021	Men	377	0	9320000	4.05	(3.63-4.45)	0.00	(0.00-1.00)
2021	Women	853	1	10358000	8.24	(7.68-8.78)	0.12	(0.02-0.67)

Supplementary Table 2. Yearly notified incidence by age groups among Chagas disease cases in Chile, 2007–2021

Year	Age	Cases	Population	Incidence	95%CI
2007	<15 years	6	16598000	0.036	0.013-0.079
2008	<15 years	11	16763000	0.066	0.033-0.117
2009	<15 years	34	16929000	0.201	0.139-0.281
2010	<15 years	17	17094000	0.099	0.058-0.159
2011	<15 years	25	17248000	0.145	0.094-0.214
2012	<15 years	29	17445000	0.166	0.111-0.239
2013	<15 years	22	17612000	0.125	0.078-0.189
2014	<15 years	20	17787000	0.112	0.069-0.174
2015	<15 years	23	17971000	0.128	0.081-0.192
2016	<15 years	25	18167000	0.138	0.089-0.203
2017	<15 years	38	18419000	0.206	0.146-0.283
2018	<15 years	42	18751000	0.224	0.161-0.303
2019	<15 years	27	19107000	0.141	0.093-0.206
2020	<15 years	20	19458000	0.103	0.063-0.159
2021	<15 years	29	19678000	0.147	0.099-0.212
2007	15-24 years	32	16598000	0.193	0.132-0.272
2008	15-24 years	44	16763000	0.262	0.191-0.352
2009	15-24 years	66	16929000	0.39	0.302-0.496
2010	15-24 years	86	17094000	0.503	0.402-0.621
2011	15-24 years	56	17248000	0.325	0.245-0.422
2012	15-24 years	46	17445000	0.264	0.193-0.352
2013	15-24 years	52	17612000	0.295	0.221-0.387
2014	15-24 years	64	17787000	0.36	0.277-0.459
2015	15-24 years	80	17971000	0.445	0.353-0.554
2016	15-24 years	109	18167000	0.6	0.493-0.724
2017	15-24 years	108	18419000	0.586	0.481-0.708
2018	15-24 years	109	18751000	0.581	0.477-0.701
2019	15-24 years	107	19107000	0.56	0.459-0.677
2020	15-24 years	55	19458000	0.283	0.213-0.368
2021	15-24 years	65	19678000	0.33	0.255-0.421
2007	25-34 years	80	16598000	0.482	0.382-0.6
2008	25-34 years	92	16763000	0.549	0.442-0.673
2009	25-34 years	136	16929000	0.803	0.674-0.95
2010	25-34 years	105	17094000	0.614	0.502-0.744
2011	25-34 years	125	17248000	0.725	0.603-0.863
2012	25-34 years	115	17445000	0.659	0.544-0.791
2013	25-34 years	94	17612000	0.534	0.431-0.653
2014	25-34 years	116	17787000	0.652	0.539-0.782
2015	25-34 years	177	17971000	0.985	0.845-1.141
2016	25-34 years	209	18167000	1.15	1-1.317
2017	25-34 years	222	18419000	1.205	1.052-1.375

2018	25-34 years	269	18751000	1.435	1.268-1.617
2019	25-34 years	224	19107000	1.172	1.024-1.336
2020	25-34 years	176	19458000	0.905	0.776-1.048
2021	25-34 years	285	19678000	1.448	1.285-1.627
2007	35-44 years	77	16598000	0.464	0.366-0.58
2008	35-44 years	133	16763000	0.793	0.664-0.94
2009	35-44 years	217	16929000	1.282	1.117-1.464
2010	35-44 years	195	17094000	1.141	0.986-1.313
2011	35-44 years	286	17248000	1.658	1.472-1.862
2012	35-44 years	190	17445000	1.089	0.94-1.255
2013	35-44 years	132	17612000	0.749	0.627-0.889
2014	35-44 years	154	17787000	0.866	0.734-1.014
2015	35-44 years	185	17971000	1.029	0.886-1.189
2016	35-44 years	183	18167000	1.007	0.867-1.164
2017	35-44 years	199	18419000	1.08	0.936-1.241
2018	35-44 years	238	18751000	1.269	1.113-1.441
2019	35-44 years	166	19107000	0.869	0.742-1.011
2020	35-44 years	113	19458000	0.581	0.479-0.698
2021	35-44 years	185	19678000	0.94	0.81-1.086
2007	45-54 years	89	16598000	0.536	0.431-0.66
2008	45-54 years	123	16763000	0.734	0.61-0.875
2009	45-54 years	273	16929000	1.613	1.427-1.816
2010	45-54 years	271	17094000	1.585	1.402-1.786
2011	45-54 years	483	17248000	2.8	2.556-3.062
2012	45-54 years	279	17445000	1.599	1.417-1.798
2013	45-54 years	201	17612000	1.141	0.989-1.31
2014	45-54 years	203	17787000	1.141	0.99-1.31
2015	45-54 years	249	17971000	1.386	1.219-1.569
2016	45-54 years	205	18167000	1.128	0.979-1.294
2017	45-54 years	245	18419000	1.33	1.169-1.508
2018	45-54 years	236	18751000	1.259	1.103-1.43
2019	45-54 years	215	19107000	1.125	0.98-1.286
2020	45-54 years	129	19458000	0.663	0.554-0.788
2021	45-54 years	180	19678000	0.915	0.786-1.059
2007	55-64 years	73	16598000	0.44	0.345-0.553
2008	55-64 years	86	16763000	0.513	0.41-0.634
2009	55-64 years	195	16929000	1.152	0.996-1.325
2010	55-64 years	233	17094000	1.363	1.194-1.55
2011	55-64 years	436	17248000	2.528	2.296-2.777
2012	55-64 years	313	17445000	1.794	1.601-2.004
2013	55-64 years	187	17612000	1.062	0.915-1.225
2014	55-64 years	203	17787000	1.141	0.99-1.31
2015	55-64 years	241	17971000	1.341	1.177-1.521
2016	55-64 years	243	18167000	1.338	1.175-1.517
2017	55-64 years	249	18419000	1.352	1.189-1.531

2018	55-64 years	257	18751000	1.371	1.208-1.549
2019	55-64 years	270	19107000	1.413	1.25-1.592
2020	55-64 years	122	19458000	0.627	0.521-0.749
2021	55-64 years	202	19678000	1.027	0.89-1.178
2007	65-74 years	43	16598000	0.259	0.187-0.349
2008	65-74 years	45	16763000	0.268	0.196-0.359
2009	65-74 years	100	16929000	0.591	0.481-0.718
2010	65-74 years	148	17094000	0.866	0.732-1.017
2011	65-74 years	334	17248000	1.936	1.734-2.156
2012	65-74 years	196	17445000	1.124	0.972-1.292
2013	65-74 years	151	17612000	0.857	0.726-1.006
2014	65-74 years	132	17787000	0.742	0.621-0.88
2015	65-74 years	160	17971000	0.89	0.758-1.039
2016	65-74 years	191	18167000	1.051	0.908-1.211
2017	65-74 years	182	18419000	0.988	0.85-1.143
2018	65-74 years	239	18751000	1.275	1.118-1.447
2019	65-74 years	237	19107000	1.24	1.087-1.409
2020	65-74 years	119	19458000	0.612	0.507-0.732
2021	65-74 years	145	19678000	0.737	0.622-0.867
2007	≥75 years	21	16598000	0.127	0.078-0.193
2008	≥75 years	31	16763000	0.185	0.126-0.262
2009	≥75 years	61	16929000	0.36	0.276-0.463
2010	≥75 years	79	17094000	0.462	0.366-0.576
2011	≥75 years	204	17248000	1.183	1.026-1.357
2012	≥75 years	136	17445000	0.78	0.654-0.922
2013	≥75 years	86	17612000	0.488	0.391-0.603
2014	≥75 years	91	17787000	0.512	0.412-0.628
2015	≥75 years	100	17971000	0.556	0.453-0.677
2016	≥75 years	142	18167000	0.782	0.658-0.921
2017	≥75 years	174	18419000	0.945	0.81-1.096
2018	≥75 years	191	18751000	1.019	0.879-1.174
2019	≥75 years	175	19107000	0.916	0.785-1.062
2020	≥75 years	88	19458000	0.452	0.363-0.557
2021	≥75 years	138	19678000	0.701	0.589-0.829

Supplementary Table 3. Yearly incidence and Chagas-specific mortality proportion by diagnostic stage among Chagas disease cases in Chile, 2007–2021

Year	Diagnostic stage	Population	Cases	Deceased	Incidence	95%CI	Chagas-specific mortality proportion (%)	95%CI
2007	Acute	16598000	49	0	0.30	(0.22-0.39)	0.00	(0.00-7.25)
2007	Chronic	16598000	379	11	2.28	(2.06-2.53)	2.90	(1.46-5.13)
2008	Acute	16763000	136	3	0.81	(0.68-0.96)	2.21	(0.46-6.31)
2008	Chronic	16763000	432	11	2.58	(2.34-2.83)	2.55	(1.28-4.51)
2009	Acute	16929000	281	1	1.66	(1.47-1.87)	0.36	(0.01-1.97)
2009	Chronic	16929000	808	11	4.77	(4.45-5.11)	1.36	(0.68-2.42)
2010	Acute	17094000	439	7	2.57	(2.33-2.82)	1.59	(0.64-3.26)
2010	Chronic	17094000	703	20	4.11	(3.81-4.43)	2.84	(1.75-4.36)
2011	Acute	17248000	522	12	3.03	(2.77-3.30)	2.30	(1.19-3.98)
2011	Chronic	17248000	1450	42	8.41	(7.98-8.85)	2.90	(2.10-3.90)
2012	Acute	17445000	24	1	0.14	(0.09-0.20)	4.17	(0.11-21.12)
2012	Chronic	17445000	1290	33	7.39	(7.00-7.81)	2.56	(1.77-3.57)
2013	Acute	17612000	1	1	0.01	(0.00-0.03)	100.00	(2.50-100.00)
2013	Chronic	17612000	942	21	5.35	(5.01-5.70)	2.23	(1.39-3.39)
2014	Acute	17787000	2	0	0.01	(0.00-0.04)	0.00	(0.00-84.19)
2014	Chronic	17787000	986	19	5.54	(5.20-5.90)	1.93	(1.16-2.99)
2015	Acute	17971000	2	0	0.01	(0.00-0.04)	0.00	(0.00-84.19)
2015	Chronic	17971000	1231	10	6.85	(6.47-7.24)	0.81	(0.39-1.49)
2016	Acute	18167000	1	0	0.01	(0.00-0.03)	0.00	(0.00-97.50)
2016	Chronic	18167000	1315	16	7.24	(6.85-7.64)	1.22	(0.70-1.97)
2017	Acute	18419000	2	0	0.01	(0.00-0.04)	0.00	(0.00-84.19)
2017	Chronic	18419000	1420	12	7.71	(7.31-8.12)	0.85	(0.44-1.47)
2018	Acute	18751000	7	0	0.04	(0.02-0.08)	0.00	(0.00-40.96)
2018	Chronic	18751000	1596	16	8.51	(8.10-8.94)	1.00	(0.57-1.62)
2019	Acute	19107000	25	0	0.13	(0.08-0.19)	0.00	(0.00-13.72)
2019	Chronic	19107000	1410	8	7.38	(7.00-7.77)	0.57	(0.25-1.11)
2020	Acute	19458000	27	1	0.14	(0.09-0.20)	3.70	(0.09-18.97)
2020	Chronic	19458000	800	4	4.11	(3.83-4.41)	0.50	(0.14-1.28)
2021	Acute	19678000	19	0	0.10	(0.06-0.15)	0.00	(0.00-17.65)
2021	Chronic	19678000	1211	1	6.15	(5.81-6.51)	0.08	(0.00-0.46)

Supplementary Table 4. Yearly notified incidence by ICD-10 codes among Chagas disease cases in Chile, 2007–2021

Year	ICD-10_code	Cases	Population	Incidence	95%CI
2007	B57.0	17	16598000	0.10	(0.06-0.16)
2007	B57.1	32	16598000	0.19	(0.13-0.27)
2007	B57.2	223	16598000	1.34	(1.17-1.53)
2007	B57.3	5	16598000	0.03	(0.01-0.07)
2007	B57.5	148	16598000	0.89	(0.75-1.05)
2008	B57.0	28	16763000	0.17	(0.11-0.24)
2008	B57.1	108	16763000	0.64	(0.53-0.78)
2008	B57.2	192	16763000	1.15	(0.99-1.32)
2008	B57.3	19	16763000	0.11	(0.07-0.18)
2008	B57.5	221	16763000	1.32	(1.15-1.50)
2009	B57.0	12	16929000	0.07	(0.04-0.12)
2009	B57.1	269	16929000	1.59	(1.40-1.79)
2009	B57.2	498	16929000	2.94	(2.69-3.21)
2009	B57.3	23	16929000	0.14	(0.09-0.20)
2009	B57.5	285	16929000	1.68	(1.49-1.89)
2009	Z22.8	2	16929000	0.01	(0.00-0.04)
2010	B57.0	27	17094000	0.16	(0.10-0.23)
2010	B57.1	412	17094000	2.41	(2.18-2.65)
2010	B57.2	367	17094000	2.15	(1.93-2.38)
2010	B57.3	24	17094000	0.14	(0.09-0.21)
2010	B57.5	304	17094000	1.78	(1.58-1.99)
2010	Z22.8	8	17094000	0.05	(0.02-0.09)
2011	B57.0	23	17248000	0.13	(0.08-0.20)
2011	B57.1	499	17248000	2.89	(2.64-3.16)
2011	B57.2	489	17248000	2.84	(2.59-3.10)
2011	B57.3	34	17248000	0.20	(0.14-0.28)
2011	B57.5	206	17248000	1.19	(1.04-1.37)
2011	Z22.8	717	17248000	4.16	(3.86-4.47)
2012	B57.0	7	17445000	0.04	(0.02-0.08)
2012	B57.1	17	17445000	0.10	(0.06-0.16)
2012	B57.2	167	17445000	0.96	(0.82-1.11)
2012	B57.3	30	17445000	0.17	(0.12-0.25)
2012	B57.5	191	17445000	1.09	(0.95-1.26)
2012	Z22.8	897	17445000	5.14	(4.81-5.49)
2013	B57.1	1	17612000	0.01	(0.00-0.03)
2013	B57.2	72	17612000	0.41	(0.32-0.51)
2013	B57.3	25	17612000	0.14	(0.09-0.21)
2013	B57.5	111	17612000	0.63	(0.52-0.76)
2013	Z22.8	730	17612000	4.14	(3.85-4.46)
2014	B57.0	1	17787000	0.01	(0.00-0.03)

2014	B57.1	1	17787000	0.01	(0.00-0.03)
2014	B57.2	57	17787000	0.32	(0.24-0.42)
2014	B57.3	23	17787000	0.13	(0.08-0.19)
2014	B57.5	43	17787000	0.24	(0.17-0.33)
2014	Z22.8	860	17787000	4.83	(4.52-5.17)
2015	B57.0	1	17971000	0.01	(0.00-0.03)
2015	B57.1	1	17971000	0.01	(0.00-0.03)
2015	B57.2	99	17971000	0.55	(0.45-0.67)
2015	B57.3	48	17971000	0.27	(0.20-0.35)
2015	B57.5	61	17971000	0.34	(0.26-0.44)
2015	Z22.8	1018	17971000	5.66	(5.32-6.02)
2016	B57.0	1	18167000	0.01	(0.00-0.03)
2016	B57.2	114	18167000	0.63	(0.52-0.75)
2016	B57.3	38	18167000	0.21	(0.15-0.29)
2016	B57.5	42	18167000	0.23	(0.17-0.31)
2016	Z22.8	1103	18167000	6.07	(5.72-6.44)
2017	B57.0	2	18419000	0.01	(0.00-0.04)
2017	B57.2	131	18419000	0.71	(0.59-0.84)
2017	B57.3	40	18419000	0.22	(0.16-0.30)
2017	B57.5	21	18419000	0.11	(0.07-0.17)
2017	Z22.8	1203	18419000	6.53	(6.17-6.91)
2018	B57.0	4	18751000	0.02	(0.01-0.05)
2018	B57.1	3	18751000	0.02	(0.00-0.05)
2018	B57.2	168	18751000	0.90	(0.77-1.04)
2018	B57.3	52	18751000	0.28	(0.21-0.36)
2018	B57.5	18	18751000	0.10	(0.06-0.15)
2018	Z22.8	1338	18751000	7.14	(6.76-7.53)
2019	B57.0	6	19107000	0.03	(0.01-0.07)
2019	B57.1	19	19107000	0.10	(0.06-0.16)
2019	B57.2	201	19107000	1.05	(0.91-1.21)
2019	B57.3	69	19107000	0.36	(0.28-0.46)
2019	B57.5	63	19107000	0.33	(0.25-0.42)
2019	Z22.8	1063	19107000	5.56	(5.23-5.91)
2020	B57.0	1	19458000	0.01	(0.00-0.03)
2020	B57.1	24	19458000	0.12	(0.08-0.18)
2020	B57.2	108	19458000	0.56	(0.46-0.67)
2020	B57.3	41	19458000	0.21	(0.15-0.29)
2020	B57.5	48	19458000	0.25	(0.18-0.33)
2020	Z22.8	573	19458000	2.94	(2.71-3.20)
2021	B57.0	4	19678000	0.02	(0.01-0.05)
2021	B57.1	13	19678000	0.07	(0.04-0.11)
2021	B57.2	161	19678000	0.82	(0.70-0.95)
2021	B57.3	72	19678000	0.37	(0.29-0.46)
2021	B57.5	107	19678000	0.54	(0.45-0.66)

2021	Z22.8	841	19678000	4.27	(3.99-4.57)

Notes: ICD-10 codes were used to classify Chagas disease diagnoses: Z22.8, asymptomatic carriers; B57.2, chronic cardiac form; B57.3, chronic digestive form; B57.4, chronic disease affecting nervous system; B57.5, other organ involvement; B57.1, acute forms without cardiac involvement.

Supplementary Table 5. Yearly notified incidence by geographical zone among Chagas disease cases in Chile, 2007–2021

Year	Macrozone	Cases	Population	Incidence	95%CI
2007	Central macrozone	205	16598000	1.24	(1.07-1.42)
2007	Northern macrozone	197	16598000	1.19	(1.03-1.36)
2007	Metropolitan region	26	16598000	0.16	(0.10-0.23)
2008	Central macrozone	248	16763000	1.48	(1.30-1.68)
2008	Northern macrozone	239	16763000	1.43	(1.25-1.62)
2008	Metropolitan region	81	16763000	0.48	(0.38-0.60)
2009	Central macrozone	581	16929000	3.43	(3.16-3.72)
2009	Northern macrozone	276	16929000	1.63	(1.44-1.83)
2009	Southern macrozone	4	16929000	0.02	(0.01-0.06)
2009	Metropolitan region	228	16929000	1.35	(1.18-1.53)
2010	Central macrozone	558	17094000	3.26	(3.00-3.55)
2010	Northern macrozone	367	17094000	2.15	(1.93-2.38)
2010	Southern macrozone	3	17094000	0.02	(0.00-0.05)
2010	Metropolitan region	214	17094000	1.25	(1.09-1.43)
2011	Central macrozone	1367	17248000	7.93	(7.51-8.36)
2011	Northern macrozone	429	17248000	2.49	(2.26-2.73)
2011	Southern macrozone	10	17248000	0.06	(0.03-0.11)
2011	Metropolitan region	166	17248000	0.96	(0.82-1.12)
2012	Central macrozone	784	17445000	4.49	(4.19-4.82)
2012	Northern macrozone	378	17445000	2.17	(1.95-2.40)
2012	Southern macrozone	8	17445000	0.05	(0.02-0.09)
2012	Metropolitan region	144	17445000	0.83	(0.70-0.97)
2013	Central macrozone	457	17612000	2.59	(2.36-2.84)
2013	Northern macrozone	327	17612000	1.86	(1.66-2.07)
2013	Southern macrozone	12	17612000	0.07	(0.04-0.12)
2013	Metropolitan region	147	17612000	0.83	(0.71-0.98)
2014	Central macrozone	482	17787000	2.71	(2.47-2.96)
2014	Northern macrozone	318	17787000	1.79	(1.60-2.00)
2014	Southern macrozone	7	17787000	0.04	(0.02-0.08)
2014	Metropolitan region	181	17787000	1.02	(0.87-1.18)
2015	Central macrozone	536	17971000	2.98	(2.74-3.25)
2015	Northern macrozone	380	17971000	2.11	(1.91-2.34)
2015	Southern macrozone	3	17971000	0.02	(0.00-0.05)
2015	Metropolitan region	314	17971000	1.75	(1.56-1.95)
2016	Central macrozone	633	18167000	3.48	(3.22-3.77)
2016	Northern macrozone	420	18167000	2.31	(2.10-2.54)
2016	Southern macrozone	5	18167000	0.03	(0.01-0.06)
2016	Metropolitan region	258	18167000	1.42	(1.25-1.60)
2017	Central macrozone	650	18419000	3.53	(3.26-3.81)

2017	Northern macrozone	501	18419000	2.72	(2.49-2.97)
2017	Southern macrozone	10	18419000	0.05	(0.03-0.10)
2017	Metropolitan region	261	18419000	1.42	(1.25-1.60)
2018	Central macrozone	673	18751000	3.59	(3.32-3.87)
2018	Northern macrozone	716	18751000	3.82	(3.54-4.11)
2018	Southern macrozone	6	18751000	0.03	(0.01-0.07)
2018	Metropolitan region	208	18751000	1.11	(0.96-1.27)
2019	Central macrozone	643	19107000	3.37	(3.11-3.64)
2019	Northern macrozone	663	19107000	3.47	(3.21-3.74)
2019	Southern macrozone	9	19107000	0.05	(0.02-0.09)
2019	Metropolitan region	120	19107000	0.63	(0.52-0.75)
2020	Central macrozone	331	19458000	1.7	(1.52-1.89)
2020	Northern macrozone	367	19458000	1.89	(1.70-2.09)
2020	Southern macrozone	15	19458000	0.08	(0.04-0.13)
2020	Metropolitan region	114	19458000	0.59	(0.48-0.70)
2021	Central macrozone	494	19678000	2.51	(2.29-2.74)
2021	Northern macrozone	561	19678000	2.85	(2.62-3.10)
2021	Southern macrozone	15	19678000	0.08	(0.04-0.13)
2021	Metropolitan region	160	19678000	0.81	(0.69-0.95)

Supplementary Table 6. Yearly Chagas-specific mortality proportion by age groups among Chagas disease cases in Chile, 2007–2021

Year	Category	Type	Cases	Deaths	Chagas-specific mortality proportion (%)	95%CI
2007	<15 years	Chagas-Specific	6	0	0	0-45.926
2008	<15 years	Chagas-Specific	11	0	0	0-28.491
2009	<15 years	Chagas-Specific	34	0	0	0-10.282
2010	<15 years	Chagas-Specific	17	0	0	0-19.506
2011	<15 years	Chagas-Specific	25	0	0	0-13.719
2012	<15 years	Chagas-Specific	29	0	0	0-11.944
2013	<15 years	Chagas-Specific Mortality Proportion	22	0	0	0-15.437
2014	<15 years	Chagas-Specific Mortality Proportion	20	0	0	0-16.843
2015	<15 years	Chagas-Specific Mortality Proportion	23	0	0	0-14.819
2016	<15 years	Chagas-Specific Mortality Proportion	25	0	0	0-13.719
2017	<15 years	Chagas-Specific Mortality Proportion	38	0	0	0-9.251
2018	<15 years	Chagas-Specific Mortality Proportion	42	0	0	0-8.408
2019	<15 years	Chagas-Specific Mortality Proportion	27	0	0	0-12.77
2020	<15 years	Chagas-Specific Mortality Proportion	20	0	0	0-16.843
2021	<15 years	Chagas-Specific Mortality Proportion	29	0	0	0-11.944
2007	<15 years	All-Cause Mortality Proportion	6	0	0	0-45.926
2008	<15 years	All-Cause Mortality Proportion	11	0	0	0-28.491
2009	<15 years	All-Cause Mortality Proportion	34	0	0	0-10.282
2010	<15 years	All-Cause Mortality Proportion	17	0	0	0-19.506
2011	<15 years	All-Cause Mortality Proportion	25	0	0	0-13.719
2012	<15 years	All-Cause Mortality Proportion	29	1	3.448	0.087-17.764
2013	<15 years	All-Cause Mortality Proportion	22	1	4.545	0.115-22.844
2014	<15 years	All-Cause Mortality Proportion	20	1	5	0.127-24.873
2015	<15 years	All-Cause Mortality Proportion	23	0	0	0-14.819
2016	<15 years	All-Cause Mortality Proportion	25	0	0	0-13.719
2017	<15 years	All-Cause Mortality Proportion	38	0	0	0-9.251
2018	<15 years	All-Cause Mortality Proportion	42	0	0	0-8.408
2019	<15 years	All-Cause Mortality Proportion	27	0	0	0-12.77

2020	<15 years	All-Cause Mortality Proportion	20	0	0	0-16.843
2021	<15 years	All-Cause Mortality Proportion	29	0	0	0-11.944
2007	15-24 years	Chagas-Specific Mortality Proportion	32	0	0	0-10.888
2008	15-24 years	Chagas-Specific Mortality Proportion	44	0	0	0-8.042
2009	15-24 years	Chagas-Specific Mortality Proportion	66	0	0	0-5.436
2010	15-24 years	Chagas-Specific Mortality Proportion	86	0	0	0-4.199
2011	15-24 years	Chagas-Specific Mortality Proportion	56	0	0	0-6.375
2012	15-24 years	Chagas-Specific Mortality Proportion	46	0	0	0-7.706
2013	15-24 years	Chagas-Specific Mortality Proportion	52	0	0	0-6.848
2014	15-24 years	Chagas-Specific Mortality Proportion	64	0	0	0-5.601
2015	15-24 years	Chagas-Specific Mortality Proportion	80	0	0	0-4.506
2016	15-24 years	Chagas-Specific Mortality Proportion	109	0	0	0-3.328
2017	15-24 years	Chagas-Specific Mortality Proportion	108	0	0	0-3.358
2018	15-24 years	Chagas-Specific Mortality Proportion	109	0	0	0-3.328
2019	15-24 years	Chagas-Specific Mortality Proportion	107	0	0	0-3.389
2020	15-24 years	Chagas-Specific Mortality Proportion	55	0	0	0-6.487
2021	15-24 years	Chagas-Specific Mortality Proportion	65	0	0	0-5.517
2007	15-24 years	All-Cause Mortality Proportion	32	0	0	0-10.888
2008	15-24 years	All-Cause Mortality Proportion	44	0	0	0-8.042
2009	15-24 years	All-Cause Mortality Proportion	66	0	0	0-5.436
2010	15-24 years	All-Cause Mortality Proportion	86	1	1.163	0.029-6.309
2011	15-24 years	All-Cause Mortality Proportion	56	0	0	0-6.375
2012	15-24 years	All-Cause Mortality Proportion	46	0	0	0-7.706
2013	15-24 years	All-Cause Mortality Proportion	52	0	0	0-6.848
2014	15-24 years	All-Cause Mortality Proportion	64	0	0	0-5.601
2015	15-24 years	All-Cause Mortality Proportion	80	0	0	0-4.506
2016	15-24 years	All-Cause Mortality Proportion	109	0	0	0-3.328
2017	15-24 years	All-Cause Mortality Proportion	108	0	0	0-3.358
2018	15-24 years	All-Cause Mortality Proportion	109	0	0	0-3.328
2019	15-24 years	All-Cause Mortality Proportion	107	0	0	0-3.389
2020	15-24 years	All-Cause Mortality Proportion	55	0	0	0-6.487

2021	15-24 years	All-Cause Mortality Proportion	65	0	0	0-5.517
2007	25-34 years	Chagas-Specific Mortality Proportion	80	0	0	0-4.506
2008	25-34 years	Chagas-Specific Mortality Proportion	92	0	0	0-3.93
2009	25-34 years	Chagas-Specific Mortality Proportion	136	0	0	0-2.676
2010	25-34 years	Chagas-Specific Mortality Proportion	105	0	0	0-3.452
2011	25-34 years	Chagas-Specific Mortality Proportion	125	0	0	0-2.908
2012	25-34 years	Chagas-Specific Mortality Proportion	115	0	0	0-3.157
2013	25-34 years	Chagas-Specific Mortality Proportion	94	0	0	0-3.848
2014	25-34 years	Chagas-Specific Mortality Proportion	116	0	0	0-3.13
2015	25-34 years	Chagas-Specific Mortality Proportion	177	0	0	0-2.063
2016	25-34 years	Chagas-Specific Mortality Proportion	209	0	0	0-1.75
2017	25-34 years	Chagas-Specific Mortality Proportion	222	0	0	0-1.648
2018	25-34 years	Chagas-Specific Mortality Proportion	269	0	0	0-1.362
2019	25-34 years	Chagas-Specific Mortality Proportion	224	0	0	0-1.633
2020	25-34 years	Chagas-Specific Mortality Proportion	176	0	0	0-2.074
2021	25-34 years	Chagas-Specific Mortality Proportion	285	0	0	0-1.286
2007	25-34 years	All-Cause Mortality Proportion	80	0	0	0-4.506
2008	25-34 years	All-Cause Mortality Proportion	92	2	2.174	0.264-7.633
2009	25-34 years	All-Cause Mortality Proportion	136	4	2.941	0.807-7.359
2010	25-34 years	All-Cause Mortality Proportion	105	1	0.952	0.024-5.192
2011	25-34 years	All-Cause Mortality Proportion	125	0	0	0-2.908
2012	25-34 years	All-Cause Mortality Proportion	115	1	0.87	0.022-4.75
2013	25-34 years	All-Cause Mortality Proportion	94	0	0	0-3.848
2014	25-34 years	All-Cause Mortality Proportion	116	1	0.862	0.022-4.71
2015	25-34 years	All-Cause Mortality Proportion	177	0	0	0-2.063
2016	25-34 years	All-Cause Mortality Proportion	209	0	0	0-1.75
2017	25-34 years	All-Cause Mortality Proportion	222	0	0	0-1.648
2018	25-34 years	All-Cause Mortality Proportion	269	0	0	0-1.362
2019	25-34 years	All-Cause Mortality Proportion	224	0	0	0-1.633
2020	25-34 years	All-Cause Mortality Proportion	176	0	0	0-2.074
2021	25-34 years	All-Cause Mortality Proportion	285	0	0	0-1.286

2007	35-44 years	Chagas-Specific Mortality Proportion	77	0	0	0-4.678
2008	35-44 years	Chagas-Specific Mortality Proportion	133	0	0	0-2.735
2009	35-44 years	Chagas-Specific Mortality Proportion	217	0	0	0-1.686
2010	35-44 years	Chagas-Specific Mortality Proportion	195	1	0.513	0.013-2.824
2011	35-44 years	Chagas-Specific Mortality Proportion	286	0	0	0-1.282
2012	35-44 years	Chagas-Specific Mortality Proportion	190	1	0.526	0.013-2.897
2013	35-44 years	Chagas-Specific Mortality Proportion	132	0	0	0-2.756
2014	35-44 years	Chagas-Specific Mortality Proportion	154	0	0	0-2.367
2015	35-44 years	Chagas-Specific Mortality Proportion	185	0	0	0-1.974
2016	35-44 years	Chagas-Specific Mortality Proportion	183	0	0	0-1.996
2017	35-44 years	Chagas-Specific Mortality Proportion	199	0	0	0-1.837
2018	35-44 years	Chagas-Specific Mortality Proportion	238	0	0	0-1.538
2019	35-44 years	Chagas-Specific Mortality Proportion	166	0	0	0-2.198
2020	35-44 years	Chagas-Specific Mortality Proportion	113	0	0	0-3.212
2021	35-44 years	Chagas-Specific Mortality Proportion	185	0	0	0-1.974
2007	35-44 years	All-Cause Mortality Proportion	77	0	0	0-4.678
2008	35-44 years	All-Cause Mortality Proportion	133	4	3.008	0.825-7.521
2009	35-44 years	All-Cause Mortality Proportion	217	6	2.765	1.021-5.921
2010	35-44 years	All-Cause Mortality Proportion	195	4	2.051	0.562-5.169
2011	35-44 years	All-Cause Mortality Proportion	286	6	2.098	0.774-4.51
2012	35-44 years	All-Cause Mortality Proportion	190	1	0.526	0.013-2.897
2013	35-44 years	All-Cause Mortality Proportion	132	2	1.515	0.184-5.366
2014	35-44 years	All-Cause Mortality Proportion	154	2	1.299	0.158-4.612
2015	35-44 years	All-Cause Mortality Proportion	185	1	0.541	0.014-2.975
2016	35-44 years	All-Cause Mortality Proportion	183	1	0.546	0.014-3.007
2017	35-44 years	All-Cause Mortality Proportion	199	0	0	0-1.837
2018	35-44 years	All-Cause Mortality Proportion	238	1	0.42	0.011-2.319
2019	35-44 years	All-Cause Mortality Proportion	166	0	0	0-2.198
2020	35-44 years	All-Cause Mortality Proportion	113	0	0	0-3.212
2021	35-44 years	All-Cause Mortality Proportion	185	0	0	0-1.974
2007	45-54 years	Chagas-Specific Mortality Proportion	89	2	2.247	0.273-7.883

2008	45-54 years	Chagas-Specific Mortality Proportion	123	2	1.626	0.198-5.75
2009	45-54 years	Chagas-Specific Mortality Proportion	273	1	0.366	0.009-2.024
2010	45-54 years	Chagas-Specific Mortality Proportion	271	2	0.738	0.09-2.64
2011	45-54 years	Chagas-Specific Mortality Proportion	483	3	0.621	0.128-1.804
2012	45-54 years	Chagas-Specific Mortality Proportion	279	4	1.434	0.392-3.63
2013	45-54 years	Chagas-Specific Mortality Proportion	201	0	0	0-1.819
2014	45-54 years	Chagas-Specific Mortality Proportion	203	0	0	0-1.801
2015	45-54 years	Chagas-Specific Mortality Proportion	249	1	0.402	0.01-2.217
2016	45-54 years	Chagas-Specific Mortality Proportion	205	1	0.488	0.012-2.688
2017	45-54 years	Chagas-Specific Mortality Proportion	245	0	0	0-1.494
2018	45-54 years	Chagas-Specific Mortality Proportion	236	2	0.847	0.103-3.028
2019	45-54 years	Chagas-Specific Mortality Proportion	215	0	0	0-1.701
2020	45-54 years	Chagas-Specific Mortality Proportion	129	0	0	0-2.819
2021	45-54 years	Chagas-Specific Mortality Proportion	180	0	0	0-2.029
2007	45-54 years	All-Cause Mortality Proportion	89	14	15.73	8.875-24.982
2008	45-54 years	All-Cause Mortality Proportion	123	11	8.943	4.549-15.438
2009	45-54 years	All-Cause Mortality Proportion	273	14	5.128	2.832-8.455
2010	45-54 years	All-Cause Mortality Proportion	271	8	2.952	1.283-5.734
2011	45-54 years	All-Cause Mortality Proportion	483	13	2.692	1.441-4.559
2012	45-54 years	All-Cause Mortality Proportion	279	6	2.151	0.793-4.622
2013	45-54 years	All-Cause Mortality Proportion	201	4	1.99	0.545-5.017
2014	45-54 years	All-Cause Mortality Proportion	203	4	1.97	0.539-4.968
2015	45-54 years	All-Cause Mortality Proportion	249	4	1.606	0.439-4.062
2016	45-54 years	All-Cause Mortality Proportion	205	3	1.463	0.303-4.217
2017	45-54 years	All-Cause Mortality Proportion	245	0	0	0-1.494
2018	45-54 years	All-Cause Mortality Proportion	236	4	1.695	0.464-4.283
2019	45-54 years	All-Cause Mortality Proportion	215	0	0	0-1.701
2020	45-54 years	All-Cause Mortality Proportion	129	1	0.775	0.02-4.243
2021	45-54 years	All-Cause Mortality Proportion	180	0	0	0-2.029
2007	55-64 years	Chagas-Specific Mortality Proportion	73	0	0	0-4.928
2008	55-64 years	Chagas-Specific Mortality Proportion	86	4	4.651	1.282-11.483

2009	55-64 years	Chagas-Specific Mortality Proportion	195	3	1.538	0.318-4.43
2010	55-64 years	Chagas-Specific Mortality Proportion	233	7	3.004	1.216-6.092
2011	55-64 years	Chagas-Specific Mortality Proportion	436	10	2.294	1.105-4.177
2012	55-64 years	Chagas-Specific Mortality Proportion	313	4	1.278	0.349-3.24
2013	55-64 years	Chagas-Specific Mortality Proportion	187	3	1.604	0.332-4.617
2014	55-64 years	Chagas-Specific Mortality Proportion	203	4	1.97	0.539-4.968
2015	55-64 years	Chagas-Specific Mortality Proportion	241	2	0.83	0.101-2.965
2016	55-64 years	Chagas-Specific Mortality Proportion	243	1	0.412	0.01-2.271
2017	55-64 years	Chagas-Specific Mortality Proportion	249	2	0.803	0.097-2.871
2018	55-64 years	Chagas-Specific Mortality Proportion	257	1	0.389	0.01-2.149
2019	55-64 years	Chagas-Specific Mortality Proportion	270	2	0.741	0.09-2.65
2020	55-64 years	Chagas-Specific Mortality Proportion	122	2	1.639	0.199-5.796
2021	55-64 years	Chagas-Specific Mortality Proportion	202	0	0	0-1.81
2007	55-64 years	All-Cause Mortality Proportion	73	10	13.699	6.769-23.753
2008	55-64 years	All-Cause Mortality Proportion	86	11	12.791	6.562-21.735
2009	55-64 years	All-Cause Mortality Proportion	195	35	17.949	12.832-24.068
2010	55-64 years	All-Cause Mortality Proportion	233	27	11.588	7.777-16.411
2011	55-64 years	All-Cause Mortality Proportion	436	33	7.569	5.267-10.465
2012	55-64 years	All-Cause Mortality Proportion	313	22	7.029	4.457-10.448
2013	55-64 years	All-Cause Mortality Proportion	187	16	8.556	4.97-13.523
2014	55-64 years	All-Cause Mortality Proportion	203	11	5.419	2.736-9.489
2015	55-64 years	All-Cause Mortality Proportion	241	11	4.564	2.3-8.02
2016	55-64 years	All-Cause Mortality Proportion	243	5	2.058	0.671-4.736
2017	55-64 years	All-Cause Mortality Proportion	249	6	2.41	0.889-5.171
2018	55-64 years	All-Cause Mortality Proportion	257	1	0.389	0.01-2.149
2019	55-64 years	All-Cause Mortality Proportion	270	2	0.741	0.09-2.65
2020	55-64 years	All-Cause Mortality Proportion	122	3	2.459	0.51-7.018
2021	55-64 years	All-Cause Mortality Proportion	202	0	0	0-1.81
2007	65-74 years	Chagas-Specific Mortality Proportion	43	5	11.628	3.88-25.08
2008	65-74 years	Chagas-Specific Mortality Proportion	45	3	6.667	1.397-18.268
2009	65-74 years	Chagas-Specific Mortality Proportion	100	6	6	2.233-12.603

2010	65-74 years	Chagas-Specific Mortality Proportion	148	7	4.73	1.922-9.502
2011	65-74 years	Chagas-Specific Mortality Proportion	334	21	6.287	3.934-9.451
2012	65-74 years	Chagas-Specific Mortality Proportion	196	8	4.082	1.778-7.884
2013	65-74 years	Chagas-Specific Mortality Proportion	151	7	4.636	1.884-9.318
2014	65-74 years	Chagas-Specific Mortality Proportion	132	4	3.03	0.832-7.577
2015	65-74 years	Chagas-Specific Mortality Proportion	160	3	1.875	0.388-5.382
2016	65-74 years	Chagas-Specific Mortality Proportion	191	4	2.094	0.573-5.275
2017	65-74 years	Chagas-Specific Mortality Proportion	182	3	1.648	0.341-4.741
2018	65-74 years	Chagas-Specific Mortality Proportion	239	1	0.418	0.011-2.309
2019	65-74 years	Chagas-Specific Mortality Proportion	237	1	0.422	0.011-2.328
2020	65-74 years	Chagas-Specific Mortality Proportion	119	2	1.681	0.204-5.939
2021	65-74 years	Chagas-Specific Mortality Proportion	145	0	0	0-2.51
2007	65-74 years	All-Cause Mortality Proportion	43	20	46.512	31.176-62.345
2008	65-74 years	All-Cause Mortality Proportion	45	16	35.556	21.868-51.22
2009	65-74 years	All-Cause Mortality Proportion	100	36	36	26.641-46.212
2010	65-74 years	All-Cause Mortality Proportion	148	37	25	18.253-32.775
2011	65-74 years	All-Cause Mortality Proportion	334	79	23.653	19.197-28.582
2012	65-74 years	All-Cause Mortality Proportion	196	41	20.918	15.449-27.289
2013	65-74 years	All-Cause Mortality Proportion	151	27	17.881	12.127-24.939
2014	65-74 years	All-Cause Mortality Proportion	132	20	15.152	9.507-22.426
2015	65-74 years	All-Cause Mortality Proportion	160	18	11.25	6.805-17.196
2016	65-74 years	All-Cause Mortality Proportion	191	16	8.377	4.864-13.247
2017	65-74 years	All-Cause Mortality Proportion	182	10	5.495	2.666-9.872
2018	65-74 years	All-Cause Mortality Proportion	239	7	2.929	1.186-5.941
2019	65-74 years	All-Cause Mortality Proportion	237	2	0.844	0.102-3.015
2020	65-74 years	All-Cause Mortality Proportion	119	2	1.681	0.204-5.939
2021	65-74 years	All-Cause Mortality Proportion	145	0	0	0-2.512
2007	≥75 years	Chagas-Specific Mortality Proportion	21	3	14.286	3.049-36.342
2008	≥75 years	Chagas-Specific Mortality Proportion	31	5	16.129	5.452-33.727
2009	≥75 years	Chagas-Specific Mortality Proportion	61	1	1.639	0.041-8.799
2010	≥75 years	Chagas-Specific Mortality Proportion	79	9	11.392	5.344-20.528

2011	≥75 years	Chagas-Specific Mortality Proportion	204	16	7.843	4.549-12.424
2012	≥75 years	Chagas-Specific Mortality Proportion	136	13	9.559	5.188-15.791
2013	≥75 years	Chagas-Specific Mortality Proportion	86	7	8.14	3.335-16.054
2014	≥75 years	Chagas-Specific Mortality Proportion	91	10	10.989	5.397-19.282
2015	≥75 years	Chagas-Specific Mortality Proportion	100	2	2	0.243-7.038
2016	≥75 years	Chagas-Specific Mortality Proportion	142	8	5.634	2.463-10.799
2017	≥75 years	Chagas-Specific Mortality Proportion	174	7	4.023	1.633-8.113
2018	≥75 years	Chagas-Specific Mortality Proportion	191	9	4.712	2.177-8.756
2019	≥75 years	Chagas-Specific Mortality Proportion	175	4	2.286	0.626-5.749
2020	≥75 years	Chagas-Specific Mortality Proportion	88	0	0	0-4.105
2021	≥75 years	Chagas-Specific Mortality Proportion	138	1	0.725	0.018-3.971
2007	≥75 years	All-Cause Mortality Proportion	21	15	71.429	47.82-88.71
2008	≥75 years	All-Cause Mortality Proportion	31	22	70.968	51.96-85.77
2009	≥75 years	All-Cause Mortality Proportion	61	41	67.213	53.99-78.69
2010	≥75 years	All-Cause Mortality Proportion	79	46	58.228	46.58-69.23
2011	≥75 years	All-Cause Mortality Proportion	204	102	50	42.93-57.06
2012	≥75 years	All-Cause Mortality Proportion	136	58	42.647	34.21-51.41
2013	≥75 years	All-Cause Mortality Proportion	86	36	41.86	31.30-52.99
2014	≥75 years	All-Cause Mortality Proportion	91	36	39.56	29.46-50.36
2015	≥75 years	All-Cause Mortality Proportion	100	34	34	24.82-44.15
2016	≥75 years	All-Cause Mortality Proportion	142	41	28.873	21.58-37.06
2017	≥75 years	All-Cause Mortality Proportion	174	19	10.92	6.70-16.52
2018	≥75 years	All-Cause Mortality Proportion	191	18	9.424	5.68-14.48
2019	≥75 years	All-Cause Mortality Proportion	175	7	4	1.62-8.06
2020	≥75 years	All-Cause Mortality Proportion	88	2	2.273	0.27-7.97
2021	≥75 years	All-Cause Mortality Proportion	138	1	0.725	0.018-3.971

Supplementary Table 7. Yearly Chagas-specific mortality proportion (%) by ICD-10 codes among Chagas disease cases in Chile, 2007–2021

Year	ICD10_code	Cases	Deceased	Chagas-specific mortality proportion (%)	95%CI
2007	B57.1	32	0	0.00	(0.00-10.89)
2007	B57.2	223	5	2.24	(0.73-5.15)
2007	B57.3	5	0	0.00	(0.00-52.18)
2007	B57.5	148	6	4.05	(1.50-8.61)
2008	B57.1	108	3	2.78	(0.58-7.90)
2008	B57.2	192	5	2.60	(0.85-5.97)
2008	B57.3	19	3	15.79	(3.38-39.58)
2008	B57.5	221	3	1.36	(0.28-3.92)
2009	B57.1	269	1	0.37	(0.01-2.05)
2009	B57.2	498	7	1.41	(0.57-2.87)
2009	B57.3	23	1	4.35	(0.11-21.95)
2009	B57.5	285	3	1.05	(0.22-3.05)
2009	Z22.8	2	0	0.00	(0.00-84.19)
2010	B57.1	412	5	1.21	(0.40-2.81)
2010	B57.2	367	10	2.72	(1.31-4.95)
2010	B57.3	24	2	8.33	(1.03-27.00)
2010	B57.5	304	8	2.63	(1.14-5.12)
2010	Z22.8	8	0	0.00	(0.00-36.94)
2011	B57.1	499	12	2.40	(1.25-4.16)
2011	B57.2	489	18	3.68	(2.20-5.76)
2011	B57.3	34	3	8.82	(1.86-23.68)
2011	B57.5	206	3	1.46	(0.30-4.20)
2011	Z22.8	717	18	2.51	(1.49-3.94)
2012	B57.1	17	1	5.88	(0.15-28.69)
2012	B57.2	167	5	2.99	(0.98-6.85)
2012	B57.3	30	3	10.00	(2.11-26.53)
2012	B57.5	191	5	2.62	(0.86-6.00)
2012	Z22.8	897	20	2.23	(1.37-3.42)
2013	B57.1	1	1	100.00	(2.50-100.00)
2013	B57.2	72	8	11.11	(4.92-20.72)
2013	B57.3	25	2	8.00	(0.98-26.03)
2013	B57.5	111	2	1.80	(0.22-6.36)
2013	Z22.8	730	9	1.23	(0.57-2.33)
2014	B57.1	1	0	0.00	(0.00-97.50)
2014	B57.2	57	3	5.26	(1.10-14.62)
2014	B57.3	23	2	8.70	(1.07-28.04)
2014	B57.5	43	0	0.00	(0.00-8.22)
2014	Z22.8	860	14	1.63	(0.89-2.72)
2015	B57.1	1	0	0.00	(0.00-97.50)
2015	B57.2	99	3	3.03	(0.63-8.60)

2015	B57.3	48	3	6.25	(1.31-17.20)
2015	B57.5	61	1	1.64	(0.04-8.80)
2015	Z22.8	1018	3	0.29	(0.06-0.86)
2016	B57.2	114	5	4.39	(1.44-9.94)
2016	B57.3	38	4	10.53	(2.94-24.80)
2016	B57.5	42	0	0.00	(0.00-8.41)
2016	Z22.8	1103	7	0.63	(0.26-1.30)
2017	B57.2	131	2	1.53	(0.19-5.41)
2017	B57.3	40	0	0.00	(0.00-8.81)
2017	B57.5	21	0	0.00	(0.00-16.11)
2017	Z22.8	1203	10	0.83	(0.40-1.52)
2018	B57.1	3	0	0.00	(0.00-70.76)
2018	B57.2	168	6	3.57	(1.32-7.61)
2018	B57.3	52	2	3.85	(0.47-13.21)
2018	B57.5	18	1	5.56	(0.14-27.29)
2018	Z22.8	1338	7	0.52	(0.21-1.07)
2019	B57.1	19	0	0.00	(0.00-17.65)
2019	B57.2	201	5	2.49	(0.81-5.71)
2019	B57.3	69	1	1.45	(0.04-7.81)
2019	B57.5	63	0	0.00	(0.00-5.69)
2019	Z22.8	1063	2	0.19	(0.02-0.68)
2020	B57.1	24	1	4.17	(0.11-21.12)
2020	B57.2	108	2	1.85	(0.23-6.53)
2020	B57.3	41	0	0.00	(0.00-8.60)
2020	B57.5	48	1	2.08	(0.05-11.07)
2020	Z22.8	573	1	0.17	(0.00-0.97)
2021	B57.1	13	0	0.00	(0.00-24.71)
2021	B57.2	161	1	0.62	(0.02-3.41)
2021	B57.3	72	0	0.00	(0.00-4.99)
2021	B57.5	107	0	0.00	(0.00-3.39)
2021	Z22.8	841	0	0.00	(0.00-0.44)

Notes: ICD-10 codes were used to classify Chagas disease diagnoses: Z22.8, asymptomatic carriers; B57.2, chronic cardiac form; B57.3, chronic digestive form; B57.4, chronic disease affecting nervous system; B57.5, other organ involvement; B57.1, acute forms without cardiac involvement.

Supplementary Table 8. Yearly Chagas-specific mortality proportion by hospitalisation status among Chagas disease cases in Chile, 2007–2021

Year	Hospitalised status	Cases	Deceased	Chagas-specific mortality proportion (%)	95%CI
2007	Hospitalised	16	4	25.00	(10.18-49.50)
2007	Non-hospitalised	412	7	1.70	(0.83-3.47)
2008	Hospitalised	18	4	22.22	(9.00-45.21)
2008	Non-hospitalised	550	10	1.82	(0.99-3.31)
2009	Hospitalised	39	4	10.26	(4.06-23.58)
2009	Non-hospitalised	1050	8	0.76	(0.39-1.50)
2010	Hospitalised	54	6	11.11	(5.19-22.19)
2010	Non-hospitalised	1088	21	1.93	(1.27-2.93)
2011	Hospitalised	94	14	14.89	(9.08-23.46)
2011	Non-hospitalised	1878	40	2.13	(1.57-2.89)
2012	Hospitalised	65	10	15.38	(8.58-26.06)
2012	Non-hospitalised	1249	24	1.92	(1.29-2.84)
2013	Hospitalised	41	7	17.07	(8.53-31.26)
2013	Non-hospitalised	902	15	1.66	(1.01-2.73)
2014	Hospitalised	27	4	14.81	(5.92-32.48)
2014	Non-hospitalised	961	15	1.56	(0.95-2.56)
2015	Hospitalised	37	3	8.11	(2.80-21.30)
2015	Non-hospitalised	1196	7	0.59	(0.28-1.20)
2016	Hospitalised	58	9	15.52	(8.38-26.93)
2016	Non-hospitalised	1258	7	0.56	(0.27-1.14)
2017	Hospitalised	38	2	5.26	(1.46-17.29)
2017	Non-hospitalised	1384	10	0.72	(0.39-1.32)
2018	Hospitalised	42	5	11.90	(5.19-25.00)
2018	Non-hospitalised	1561	11	0.70	(0.39-1.26)
2019	Hospitalised	43	1	2.33	(0.41-12.06)
2019	Non-hospitalised	1392	7	0.50	(0.24-1.03)
2020	Hospitalised	38	3	7.89	(2.72-20.80)
2020	Non-hospitalised	789	2	0.25	(0.07-0.92)
2021	Hospitalised	42	0	0.00	(0.00-8.38)
2021	Non-hospitalised	1188	1	0.08	(0.01-0.48)

Supplementary Table 9. Yearly Chagas-specific mortality proportion (%) by age groups among hospitalised patients with Chagas disease cases in Chile, 2007–2021

Year	Age	Hospitalised	Deceased	Chagas-specific mortality proportion (%)	95%CI
2007	<15 years	0	0		
2008	<15 years	1	0	0	0-97.5
2009	<15 years	2	0	0	0-84.189
2010	<15 years	0	0		
2011	<15 years	0	0		
2012	<15 years	1	0	0	0-97.5
2013	<15 years	0	0		
2014	<15 years	0	0		
2015	<15 years	1	0	0	0-97.5
2016	<15 years	2	0	0	0-84.189
2017	<15 years	0	0		
2018	<15 years	0	0		
2019	<15 years	0	0		
2020	<15 years	1	0	0	0-97.5
2021	<15 years	0	0		
2007	15-24 years	0	0		
2008	15-24 years	2	0	0	0-84.189
2009	15-24 years	1	0	0	0-97.5
2010	15-24 years	2	0	0	0-84.189
2011	15-24 years	3	0	0	0-70.76
2012	15-24 years	0	0		
2013	15-24 years	2	0	0	0-84.189
2014	15-24 years	0	0		
2015	15-24 years	1	0	0	0-97.5
2016	15-24 years	2	0	0	0-84.189
2017	15-24 years	1	0	0	0-97.5
2018	15-24 years	2	0	0	0-84.189
2019	15-24 years	7	0	0	0-40.962
2020	15-24 years	3	0	0	0-70.76
2021	15-24 years	2	0	0	0-84.189
2007	25-34 years	0	0		
2008	25-34 years	0	0		
2009	25-34 years	1	0	0	0-97.5
2010	25-34 years	4	0	0	0-60.236
2011	25-34 years	1	0	0	0-97.5
2012	25-34 years	1	0	0	0-97.5
2013	25-34 years	1	0	0	0-97.5
2014	25-34 years	1	0	0	0-97.5
2015	25-34 years	1	0	0	0-97.5

2016	25-34 years	5	0	0	0-52.182
2017	25-34 years	5	0	0	0-52.182
2018	25-34 years	3	0	0	0-70.76
2019	25-34 years	9	0	0	0-33.627
2020	25-34 years	9	0	0	0-33.627
2021	25-34 years	9	0	0	0-33.627
2007	35-44 years	1	0	0	0-97.5
2008	35-44 years	0	0		
2009	35-44 years	5	0	0	0-52.182
2010	35-44 years	3	1	33.333	0.84-90.57
2011	35-44 years	5	0	0	0-52.182
2012	35-44 years	6	0	0	0-45.926
2013	35-44 years	2	0	0	0-84.189
2014	35-44 years	2	0	0	0-84.189
2015	35-44 years	2	0	0	0-84.189
2016	35-44 years	7	0	0	0-40.962
2017	35-44 years	2	0	0	0-84.189
2018	35-44 years	4	0	0	0-60.236
2019	35-44 years	3	0	0	0-70.76
2020	35-44 years	4	0	0	0-60.236
2021	35-44 years	6	0	0	0-45.926
2007	45-54 years	2	1	50	1.258-98.742
2008	45-54 years	5	1	20	0.505-71.642
2009	45-54 years	11	1	9.091	0.23-41.278
2010	45-54 years	8	1	12.5	0.316-52.651
2011	45-54 years	10	0	0	0-30.85
2012	45-54 years	8	1	12.5	0.316-52.651
2013	45-54 years	7	0	0	0-40.962
2014	45-54 years	5	0	0	0-52.182
2015	45-54 years	6	1	16.667	0.421-64.123
2016	45-54 years	4	1	25	0.631-80.588
2017	45-54 years	8	0	0	0-36.942
2018	45-54 years	3	2	66.667	9.43-99.16
2019	45-54 years	4	0	0	0-60.236
2020	45-54 years	6	0	0	0-45.926
2021	45-54 years	4	0	0	0-60.236
2007	55-64 years	7	0	0	0-40.962
2008	55-64 years	4	2	50	6.759-93.241
2009	55-64 years	8	0	0	0-36.942
2010	55-64 years	18	1	5.556	0.141-27.294
2011	55-64 years	22	4	18.182	5.187-40.285
2012	55-64 years	12	1	8.333	0.211-38.48
2013	55-64 years	8	1	12.5	0.316-52.651
2014	55-64 years	11	1	9.091	0.23-41.278
2015	55-64 years	6	0	0	0-45.926

2016	55-64 years	13	1	7.692	0.195-36.03
2017	55-64 years	7	0	0	0-40.962
2018	55-64 years	4	0	0	0-60.236
2019	55-64 years	5	1	20	0.505-71.642
2020	55-64 years	6	2	33.333	4.327-77.722
2021	55-64 years	9	0	0	0-33.627
2007	65-74 years	2	1	50	1.258-98.742
2008	65-74 years	5	1	20	0.505-71.642
2009	65-74 years	6	2	33.333	4.327-77.722
2010	65-74 years	13	1	7.692	0.195-36.03
2011	65-74 years	27	4	14.815	4.189-33.731
2012	65-74 years	20	3	15	3.207-37.893
2013	65-74 years	13	2	15.385	1.921-45.447
2014	65-74 years	5	1	20	0.505-71.642
2015	65-74 years	11	1	9.091	0.23-41.278
2016	65-74 years	13	1	7.692	0.195-36.03
2017	65-74 years	10	1	10	0.253-44.502
2018	65-74 years	13	0	0	0-24.705
2019	65-74 years	7	0	0	0-40.962
2020	65-74 years	5	0	0	0-52.182
2021	65-74 years	3	0	0	0-70.76
2007	≥75 years	2	1	50	1.258-98.742
2008	≥75 years	1	0	0	0-97.5
2009	≥75 years	5	1	20	0.505-71.642
2010	≥75 years	6	2	33.333	4.327-77.722
2011	≥75 years	22	4	18.182	5.187-40.285
2012	≥75 years	15	3	20	4.331-48.089
2013	≥75 years	8	4	50	15.701-84.29
2014	≥75 years	3	2	66.667	9.43-99.16
2015	≥75 years	7	0	0	0-40.962
2016	≥75 years	11	5	45.455	16.74-76.621
2017	≥75 years	5	1	20	0.505-71.642
2018	≥75 years	12	2	16.667	2.086-48.414
2019	≥75 years	8	0	0	0-36.942
2020	≥75 years	3	0	0	0-70.76
2021	≥75 years	9	0	0	0-33.627

Supplementary Table 10. Yearly Chagas-specific mortality proportion (%) by sex among hospitalised patients with Chagas disease cases in Chile, 2007–2021

Year	Sex	Hospitalised	Deceased	Chagas-specific mortality proportion (%)	95%CI
2007	Men	8	2	25.00	(3.19-65.09)
2007	Women	8	2	25.00	(3.19-65.09)
2008	Men	8	2	25.00	(3.19-65.09)
2008	Women	10	2	20.00	(2.52-55.61)
2009	Men	22	3	13.64	(2.91-34.91)
2009	Women	17	1	5.88	(0.15-28.69)
2010	Men	26	4	15.38	(4.36-34.87)
2010	Women	28	2	7.14	(0.88-23.50)
2011	Men	60	9	15.00	(7.10-26.57)
2011	Women	34	5	14.71	(4.95-31.06)
2012	Men	36	8	22.22	(10.12-39.15)
2012	Women	29	2	6.90	(0.85-22.77)
2013	Men	18	2	11.11	(1.38-34.71)
2013	Women	23	5	21.74	(7.46-43.70)
2014	Men	17	3	17.65	(3.80-43.43)
2014	Women	10	1	10.00	(0.25-44.50)
2015	Men	21	2	9.52	(1.17-30.38)
2015	Women	16	1	6.25	(0.16-30.23)
2016	Men	31	6	19.35	(7.45-37.47)
2016	Women	27	3	11.11	(2.35-29.16)
2017	Men	21	2	9.52	(1.17-30.38)
2017	Women	17	0	0.00	(0.00-19.51)
2018	Men	27	2	7.41	(0.91-24.29)
2018	Women	15	3	20.00	(4.33-48.09)
2019	Men	9	0	0.00	(0.00-33.63)
2019	Women	34	1	2.94	(0.07-15.33)
2020	Men	12	3	25.00	(5.49-57.19)
2020	Women	26	0	0.00	(0.00-13.23)
2021	Men	20	0	0.00	(0.00-16.84)
2021	Women	22	0	0.00	(0.00-15.44)

Supplementary Table 11. Yearly Chagas-specific mortality proportion (%) by diagnostic stage among hospitalised patients with Chagas disease cases in Chile, 2007–2021

Year	Diagnostic stage	Hospitalised	Deceased	Chagas-specific mortality proportion (%)	95%CI
2007	Acute	1	0	0.00	(0.00-97.50)
2007	Chronic	15	4	26.67	(7.79-55.10)
2008	Acute	4	1	25.00	(0.63-80.59)
2008	Chronic	14	3	21.43	(4.66-50.80)
2009	Acute	7	0	0.00	(0.00-40.96)
2009	Chronic	32	4	12.50	(3.51-28.99)
2010	Acute	16	1	6.25	(0.16-30.23)
2010	Chronic	38	5	13.16	(4.41-28.09)
2011	Acute	17	2	11.76	(1.46-36.44)
2011	Chronic	77	12	15.58	(8.32-25.64)
2012	Acute	1	0	0.00	(0.00-97.50)
2012	Chronic	64	10	15.62	(7.76-26.86)
2013	Acute	1	1	100.00	(2.50-100.00)
2013	Chronic	40	6	15.00	(5.71-29.84)
2014	Acute	1	0	0.00	(0.00-97.50)
2014	Chronic	26	4	15.38	(4.36-34.87)
2015	Chronic	37	3	8.11	(1.70-21.91)
2016	Chronic	58	9	15.52	(7.35-27.42)
2017	Chronic	38	2	5.26	(0.64-17.75)
2018	Chronic	42	5	11.90	(3.98-25.63)
2019	Acute	1	0	0.00	(0.00-97.50)
2019	Chronic	42	1	2.38	(0.06-12.57)
2020	Acute	2	0	0.00	(0.00-84.19)
2020	Chronic	36	3	8.33	(1.75-22.47)
2021	Chronic	42	0	0.00	(0.00-8.41)

Supplementary Table 12. Yearly Chagas-specific mortality proportion by ICD-10 codes among hospitalised patients with Chagas disease cases in Chile, 2007–2021

Year	ICD-10 code	Hospitalised	Deceased	Chagas-specific mortality proportion (%)	95%CI
2007	B57.1	1	0	0.00	(0.00-97.50)
2007	B57.2	8	2	25.00	(3.19-65.09)
2007	B57.3	2	0	0.00	(0.00-84.19)
2007	B57.5	5	2	40.00	(5.27-85.34)
2008	B57.1	4	1	25.00	(0.63-80.59)
2008	B57.2	11	3	27.27	(6.02-60.97)
2008	B57.3	1	0	0.00	(0.00-97.50)
2008	B57.5	2	0	0.00	(0.00-84.19)
2009	B57.1	7	0	0.00	(0.00-40.96)
2009	B57.2	22	2	9.09	(1.12-29.16)
2009	B57.3	2	0	0.00	(0.00-84.19)
2009	B57.5	8	2	25.00	(3.19-65.09)
2010	B57.1	14	0	0.00	(0.00-23.16)
2010	B57.2	20	2	10.00	(1.23-31.70)
2010	B57.3	3	0	0.00	(0.00-70.76)
2010	B57.5	14	3	21.43	(4.66-50.80)
2010	Z22.8	1	0	0.00	(0.00-97.50)
2011	B57.1	17	2	11.76	(1.46-36.44)
2011	B57.2	29	3	10.34	(2.19-27.35)
2011	B57.3	10	2	20.00	(2.52-55.61)
2011	B57.5	4	2	50.00	(6.76-93.24)
2011	Z22.8	34	5	14.71	(4.95-31.06)
2012	B57.1	1	0	0.00	(0.00-97.50)
2012	B57.2	5	0	0.00	(0.00-52.18)
2012	B57.3	9	2	22.22	(2.81-60.01)
2012	B57.5	10	1	10.00	(0.25-44.50)
2012	Z22.8	40	7	17.50	(7.34-32.78)
2013	B57.1	1	1	100.00	(2.50-100.00)
2013	B57.2	6	3	50.00	(11.81-88.19)
2013	B57.3	5	0	0.00	(0.00-52.18)
2013	B57.5	7	0	0.00	(0.00-40.96)
2013	Z22.8	22	3	13.64	(2.91-34.91)
2014	B57.1	1	0	0.00	(0.00-97.50)
2014	B57.2	1	0	0.00	(0.00-97.50)
2014	B57.3	5	1	20.00	(0.51-71.64)
2014	Z22.8	20	3	15.00	(3.21-37.89)
2015	B57.2	7	0	0.00	(0.00-40.96)
2015	B57.3	10	3	30.00	(6.67-65.25)
2015	B57.5	2	0	0.00	(0.00-84.19)

2015	Z22.8	18	0	0.00	(0.00-18.53)
2016	B57.2	8	2	25.00	(3.19-65.09)
2016	B57.3	11	2	18.18	(2.28-51.78)
2016	B57.5	2	0	0.00	(0.00-84.19)
2016	Z22.8	35	5	14.29	(4.81-30.26)
2017	B57.2	4	0	0.00	(0.00-60.24)
2017	B57.3	8	0	0.00	(0.00-36.94)
2017	B57.5	1	0	0.00	(0.00-97.50)
2017	Z22.8	25	2	8.00	(0.98-26.03)
2018	B57.2	12	2	16.67	(2.09-48.41)
2018	B57.3	6	1	16.67	(0.42-64.12)
2018	B57.5	1	1	100.00	(2.50-100.00)
2018	Z22.8	23	1	4.35	(0.11-21.95)
2019	B57.2	5	0	0.00	(0.00-52.18)
2019	B57.3	2	0	0.00	(0.00-84.19)
2019	B57.5	3	0	0.00	(0.00-70.76)
2019	Z22.8	32	1	3.12	(0.08-16.22)
2020	B57.1	2	0	0.00	(0.00-84.19)
2020	B57.2	9	1	11.11	(0.28-48.25)
2020	B57.3	5	0	0.00	(0.00-52.18)
2020	B57.5	1	1	100.00	(2.50-100.00)
2020	Z22.8	21	1	4.76	(0.12-23.82)
2021	B57.2	14	0	0.00	(0.00-23.16)
2021	B57.3	4	0	0.00	(0.00-60.24)
2021	B57.5	1	0	0.00	(0.00-97.50)
2021	Z22.8	22	0	0.00	(0.00-15.44)

Notes: ICD-10 codes were used to classify Chagas disease diagnoses: Z22.8, asymptomatic carriers; B57.2, chronic cardiac form; B57.3, chronic digestive form; B57.4, chronic disease affecting nervous system; B57.5, other organ involvement; B57.1, acute form without cardiac involvement.

Supplementary Table 13. Five- and ten-year cumulative incidence of Chagas-specific and other-cause death by age group.

Age group	n	Censored	Chagas deaths	Other-cause deaths	5-year Chagas-specific CIF (%)	10-year Chagas-specific CIF (%)	5-year other-cause CIF (%)	10-year other-cause CIF (%)
<15 years	368	365	0	3	0.00	0.00	0.61	1.10
15–24 years	1,079	1,078	0	1	0.00	0.00	0.09	0.09
25–34 years	2,425	2,416	0	9	0.00	0.00	0.31	0.67
35–44 years	2,653	2,625	2	26	0.05	0.05	0.80	1.27
45–54 years	3,381	3,295	18	68	0.37	0.64	1.33	2.83
55–64 years	3,310	3,117	45	148	1.05	1.73	3.39	6.35
65–74 years	2,422	2,091	75	256	2.34	4.80	8.64	15.88
≥75 years	1,717	1,239	95	383	5.26	8.89	18.90	37.23

Notes: CIF: cumulative incidence function. Age was defined at notification and grouped into predefined age bands. Chagas-specific death and other-cause death were analysed as competing events. Values are expressed as percentages and represent the estimated cumulative incidence at 5 and 10 years after notification. Censored individuals were those who remained alive without a recorded death event during follow-up.

Supplementary Table 14. Sequential multivariable Fine-Gray subdistribution hazards models for Chagas-specific mortality in notified patients with Chagas disease, Chile 2007–2021.

Models	sHR	95%CI	P-Value
**Model 1**			
Men	1.89	1.46-2.45	<0.005
**Model 2: Model 1 + Age (Per 10-year increase)**			
Men	1.49	1.15-1.94	0.003
Per 10-year increase	2.31	2.12-2.51	<0.005
**Model 3: Model 2 + Geographic zone**			
Men	1.49	1.15-1.93	0.003
Per 10-year increase	2.32	2.12-2.53	<0.005
Central macrozone	0.96	0.61-1.51	0.840
Northern macrozone	1.03	0.64-1.66	0.910
Southern macrozone	2.66	0.37-19.2	0.330
**Model 4: Model 3 + Diagnostic stage**			
Men	1.48	1.14-1.93	<0.005
Per 10-year increase	2.33	2.13-2.55	<0.005
Central macrozone	1.04	0.63-1.74	0.870
Northern macrozone	1.12	0.66-1.91	0.670
Southern macrozone	2.92	0.4-21.22	0.290

Acute stage	1.36	0.83-2.22	0.220
**Model 5: Model 4 + ICD-10 codes**			
Men	1.47	1.13-1.91	0.004
Per 10-year increase	2.31	2.11-2.53	<0.005
Central macrozone	1.08	0.65-1.79	0.780
Northern macrozone	1.07	0.63-1.82	0.820
Southern macrozone	2.55	0.33-19.7	0.370
Acute stage	2.05	1.21-3.46	0.007
B57.2	1.92	1.40-2.64	<0.005
B57.3	3.37	2.09-5.43	<0.005
B57.5	2.00	1.31-3.05	<0.005

Notes: ICD-10 codes were used to classify Chagas disease diagnoses: Z22.8, asymptomatic carriers; B57.2, chronic cardiac form; B57.3, chronic digestive form; B57.4, chronic disease affecting nervous system; B57.5, other organ involvement; B57.1, acute form without cardiac involvement.

Interactions were not pursued in Fine–Gray models to preserve parsimony and stability given the limited number of Chagas-specific deaths and sparse strata across multi-level covariates; effect modification was explored instead in complementary cause-specific Cox models incorporating time-updated hospitalisation

Supplementary Table 15. Multivariable cause-specific Cox regression models for Chagas-specific mortality in notified patients with Chagas disease, Chile 2007–2021

Models	HR	95%CI	P-Value	AIC
**Model 1: Basic model**				
Age: per 10-year increase	2.49	2.27-2.73	<0.001	3454,17
Men	1.52	1.04-2.24	0.032	
Central macrozone	0.70	0.57-0.87	0.001	
Northern macrozone	0.72	0.50-1.03	0.071	
Southern macrozone	1.95	0.32-11.8	0.466	
B57.2	1.89	1.51-2.38	<0.001	
B57.3	3.25	2.00-5.27	<0.001	
B57.5	1.79	1.06-3.02	0.030	
**Model 2: Any hospitalisation (time-fixed)**				
Age: per 10-year increase	2.46	2.23-2.71	<0.001	3385,28
Men	1.37	0.96-1.96	0.081	
Central macrozone	0.74	0.59-0.92	0.008	
Northern macrozone	0.78	0.56-1.10	0.154	
Southern macrozone	2.48	0.43-14.2	0.308	
B57.2	1.78	1.42-2.22	<0.001	
B57.3	2.54	1.79-3.62	<0.001	
B57.5	1.76	1.06-2.92	0.029	
Any hospitalisation	5.53	4.14-7.38	<0.001	
**Model 3: Number of hospitalisations (time-fixed)**				
Age: per 10-year increase	2.47	2.24-2.72	<0.001	3378,66
Men	1.33	0.92-1.92	0.123	
Central macrozone	0.74	0.59-0.92	0.007	
Northern macrozone	0.78	0.56-1.11	0.168	
Southern macrozone	2.50	0.43-14.4	0.304	
B57.2	1.73	1.42-2.11	<0.001	
B57.3	2.68	1.89-3.80	<0.001	
B57.5	1.75	1.05-2.92	0.031	
1 hospitalisation	4.28	2.86-6.40	<0.001	
2 hospitalisations	9.76	6.45-14.8	<0.001	
3+ hospitalisation	20.3	10.3-40.2	<0.001	
**Model 4: Post-hospitalisation status (time-varying)**				
Age: per 10-year increase	2.40	2.18-2.65	<0.001	3330,75
Men	1.32	0.94-1.83	0.105	
Central macrozone	0.74	0.58-0.93	0.010	
Northern macrozone	0.79	0.55-1.13	0.202	
Southern macrozone	2.41	0.43-13.2	0.316	
B57.2	1.81	1.45-2.25	<0.001	
B57.3	2.36	1.76-3.15	<0.001	
B57.5	1.90	1.12-3.20	0.016	

Post hospitalisation period	12.09	8.81-16.6	<0.001	
**Model 5: Cumulative number of hospitalisations (time-varying)**				
Age: per 10-year increase	2.43	2.21-2.68	<0.001	3319,73
Men	1.30	0.93-1.81	0.128	
Central macrozone	0.75	0.59-0.96	0.021	
Northern macrozone	0.79	0.55-1.15	0.219	
Southern macrozone	2.47	0.44-13.8	0.303	
B57.2	1.77	1.44-2.19	<0.001	
B57.3	2.45	1.82-3.31	<0.001	
B57.5	1.90	1.11-3.25	0.019	
1 hospitalisation	9.36	5.63-15.6	<0.001	
2 hospitalisations	19.2	10.4-35.9	<0.001	
3+ hospitalisation	83.9	40.9-172.2	<0.001	

Notes: Hospitalisation was evaluated under five alternative specifications: (i) Base model, excluding hospitalisation; (ii) Any hospitalisation (time-fixed), a binary indicator of ≥1 admission at any time during follow-up; (iii) Number of hospitalisations (time-fixed), categorised as 0, 1, 2, or ≥3 admissions per individual; (iv) Post-hospitalisation status (time-varying), a time-updated indicator switching from 0 to 1 at the first admission date and remaining 1 thereafter; and (v) Cumulative number of hospitalisations (time-varying), a time-updated categorical variable (0, 1, 2, ≥3) updated at each admission using a start–stop (counting-process) structure.

HR = hazard ratio; CI = confidence interval; AIC = Akaike information criterion. ICD-10 codes were used to classify Chagas disease diagnoses: Z22.8, asymptomatic carriers; B57.2, chronic cardiac form; B57.3, chronic digestive form; B57.4, chronic disease affecting nervous system; B57.5, other organ involvement; B57.1, acute form without cardiac involvement. Models 8 and 9, hospitalisation as a time-varying variable. Cum_hosp, Cumulative number of completed hospitalisations (time-updated).

Supplementary Table 16. Cox model assumption testing.

item	details
Outcome	Chagas-specific death (event_cs=1). Time origin = notification date.
Model family	Cause-specific Cox proportional hazards models.
Censoring	Other-cause deaths are censored at their time of death; alive at end follow-up censored.
Cluster-robust SE	Robust SE clustered at regional level (cluster(region)).
Hospitalisation (fixed)	hosp_any = ever hospitalised (yes/no); hosp_cat0 = 0/1/2/3+ hospitalisations.
Hospitalisation (time-varying, simple)	hosp_post = 0 before first hospitalisation, 1 after first hospitalisation.
Hospitalisation (time-varying, multiple)	hosp_cat_tv(t) = 0/1/2/3+ accumulated hospitalisations over time (start–stop).
Note on B57.1	B57.1 excluded from adjusted models due to non-estimable coefficient / sparse events.

model	n	events	AIC	Cindex	Cindex_se	PH_global_p
BASE	15664	209	3454.17	0.860	0.011	0.201
BASE + hosp_any (fixed)	15664	209	3385.28	0.879	0.013	0.148
BASE + hosp_cat0 (fixed)	15664	209	3378.66	0.880	0.013	0.236
BASE + hosp_post (time-varying)	16039	209	3330.75	0.888	0.012	0.027
BASE + hosp_cat_tv (time-varying)	16131	209	3319.73	0.888	0.013	0.037

Notes: Model fit and discrimination improved after including hospitalization variables (lower AIC and higher C-index vs. BASE). The time-varying categorical hospitalization model (hosp_cat_tv) showed the best overall performance (AIC=3319.73; C-index=0.888). Proportional hazards assumptions were assessed using global Schoenfeld residual tests; fixed-effect models showed no evidence of violation (p>0.05), while hosp_post (time-varying) indicated non-proportionality (p=0.034) and hosp_cat_tv was borderline (p=0.057).

Section 5. Supplementary figures

Supplementary Figure 1. Annual Chagas-specific and all-cause mortality proportion (%) by sex and notification year, Chile, 2007–2021.

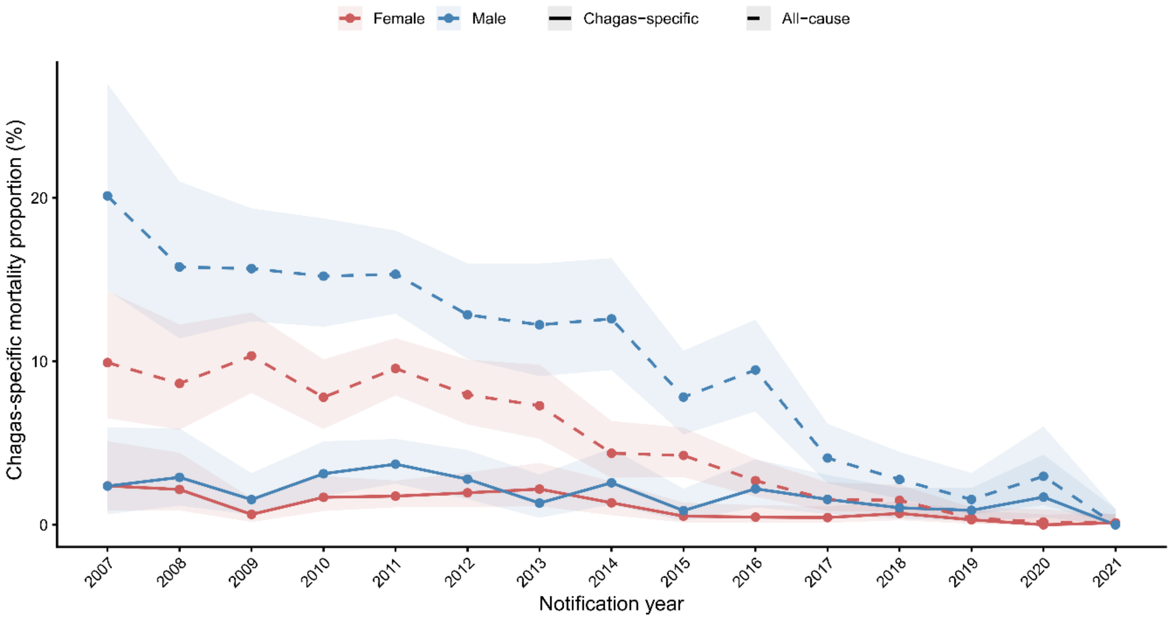

Notes:
[a] Annual Chagas-specific and all-cause mortality proportion represent the proportion of individuals notified in each calendar year who died during the available follow-up.
[b] Solid lines represent Chagas-specific and all-cause mortality proportions, defined using Chagas disease as the underlying cause of death; dashed lines represent all-cause mortality proportions, including both Chagas-specific and other-cause deaths.
[c] Red and blue lines represent women and men, respectively; shaded areas indicate 95% confidence intervals.
[d] These are crude descriptive proportions rather than cumulative incidence estimates. Comparisons across notification years should be interpreted cautiously because individuals notified in later years had shorter available follow-up, particularly in 2021.

Supplementary Figure 2. Notified incidence and Annual Chagas-specific and all-cause mortality proportion (%) of Chagas disease in Chile from 2007 to 2021, stratified by age group.

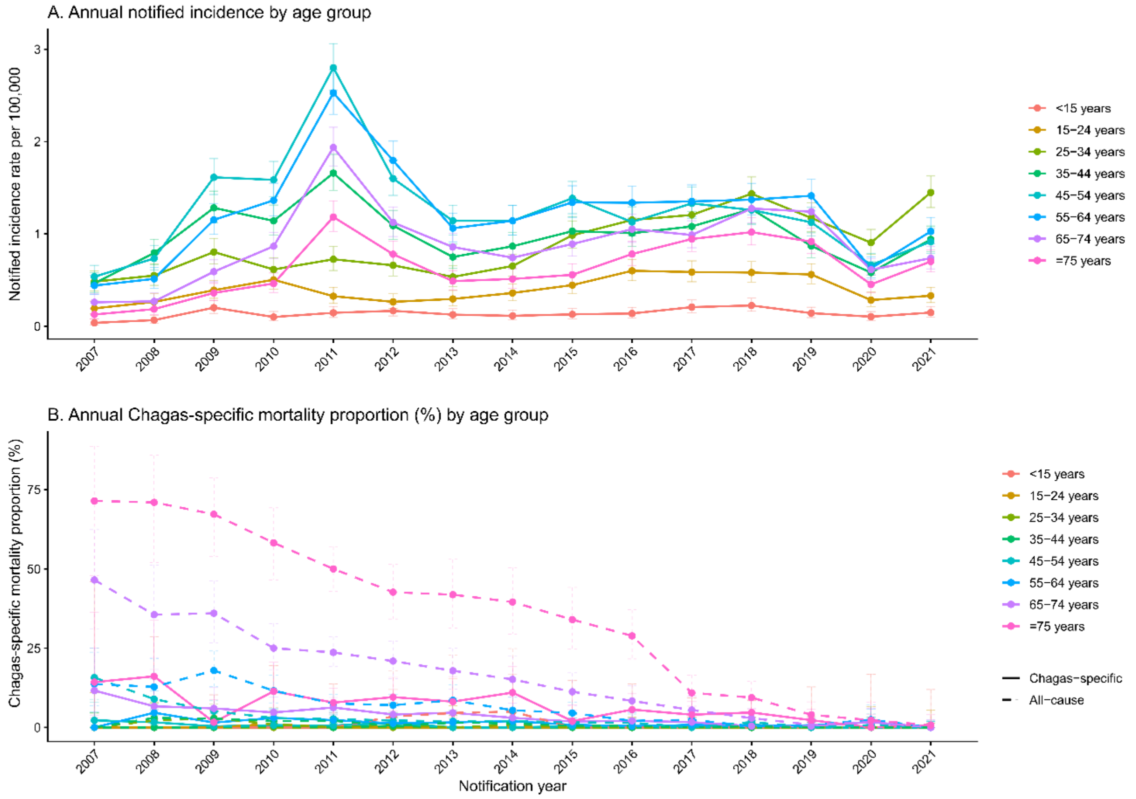

Notes:
[a] (A). Annual notified incidence rate standardised per 100,000 population, stratified by age interval
[b] (B). Annual Chagas-specific and all-cause mortality proportion (%), stratified by age group.

Supplementary Figure 3. Notified incidence and Annual Chagas-specific and all-cause mortality proportion (%) of Chagas disease in Chile from 2007 to 2021, stratified by diagnostic stage.

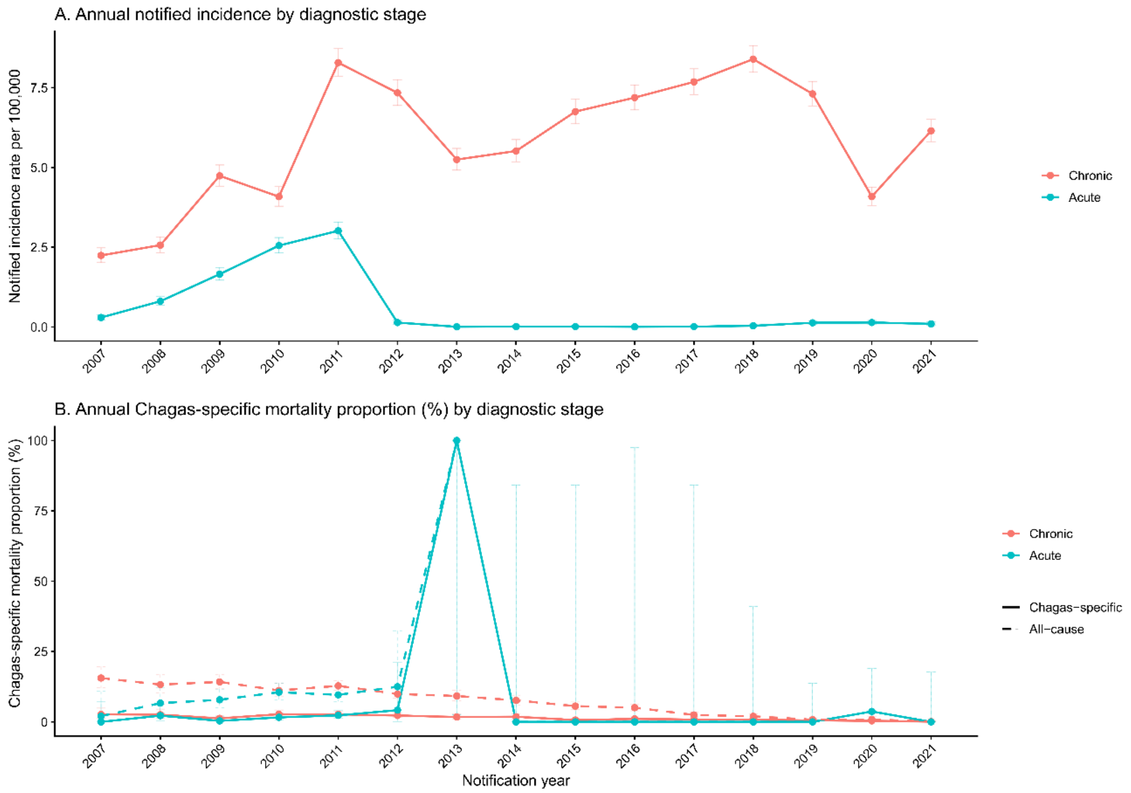

[a] (A). Annual notified incidence rate standardised per 100,000 population, stratified by diagnostic stage
[b] (B). Annual Chagas-specific and all-cause mortality proportion (%) , stratified by diagnostic stage

Supplementary Figure 4. Notified incidence and Annual Chagas-specific and all-cause mortality proportion (%) of Chagas disease in Chile from 2007 to 2021, stratified by ICD-10 category.

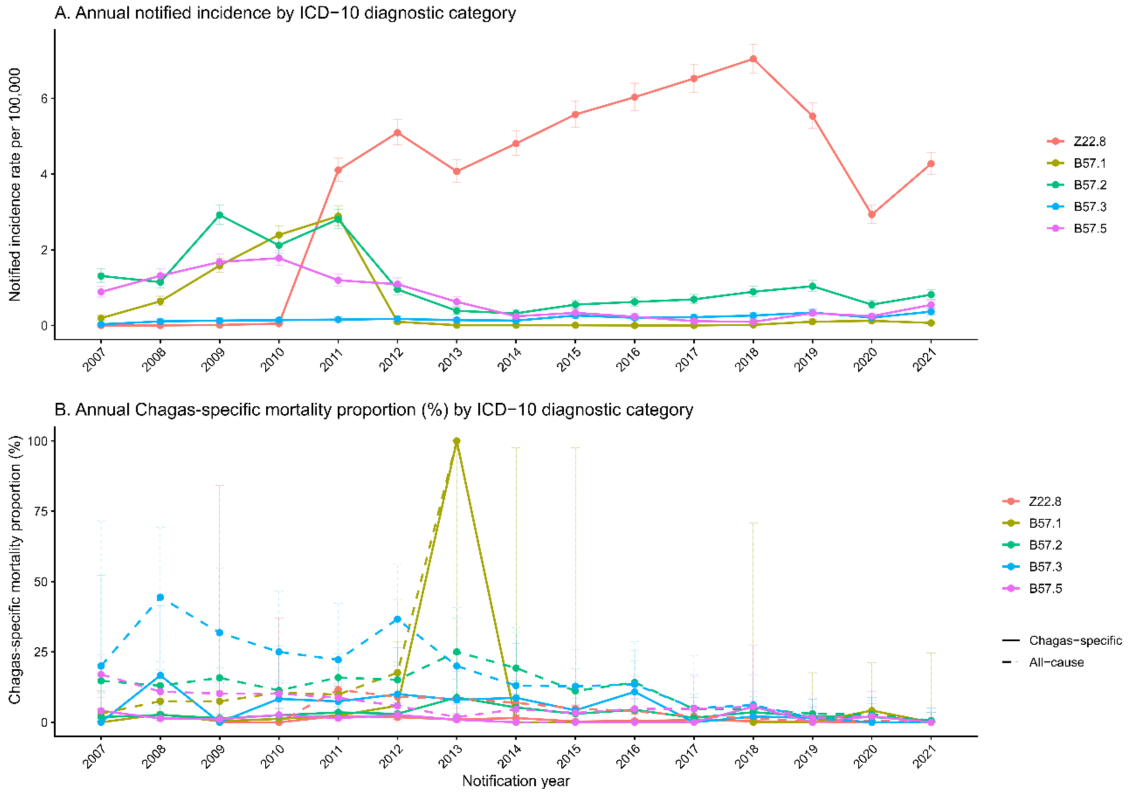

Notes

[a] (A). Annual notified incidence rate standardised per 100,000 population, stratified by ICD-10 category

[b] (B). Annual Chagas-specific and all-cause mortality proportion (%), stratified by ICD-10 category

Supplementary Figure 5. Notified incidence and Annual Chagas-specific and all-cause mortality proportion (%) of Chagas disease in Chile from 2007 to 2021, stratified by geographic zones

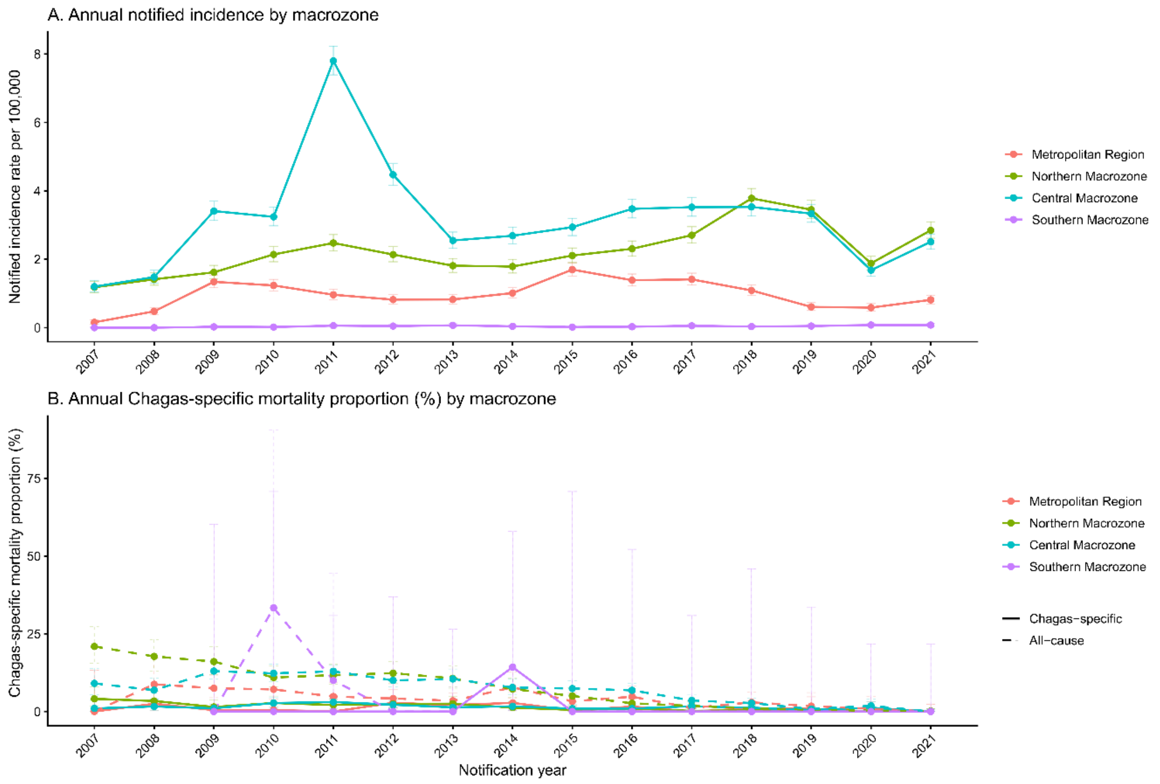

Notes

[a] (A). Annual notified incidence rate standardised per 100,000 population, stratified by geographic zones

[b] (B). Annual Chagas-specific and all-cause mortality proportion (%), stratified by geographic zones

Supplementary Figure 6. Annual Chagas-specific and all-cause mortality proportion (%) by hospitalisation status and notification year, Chile, 2007–2021.

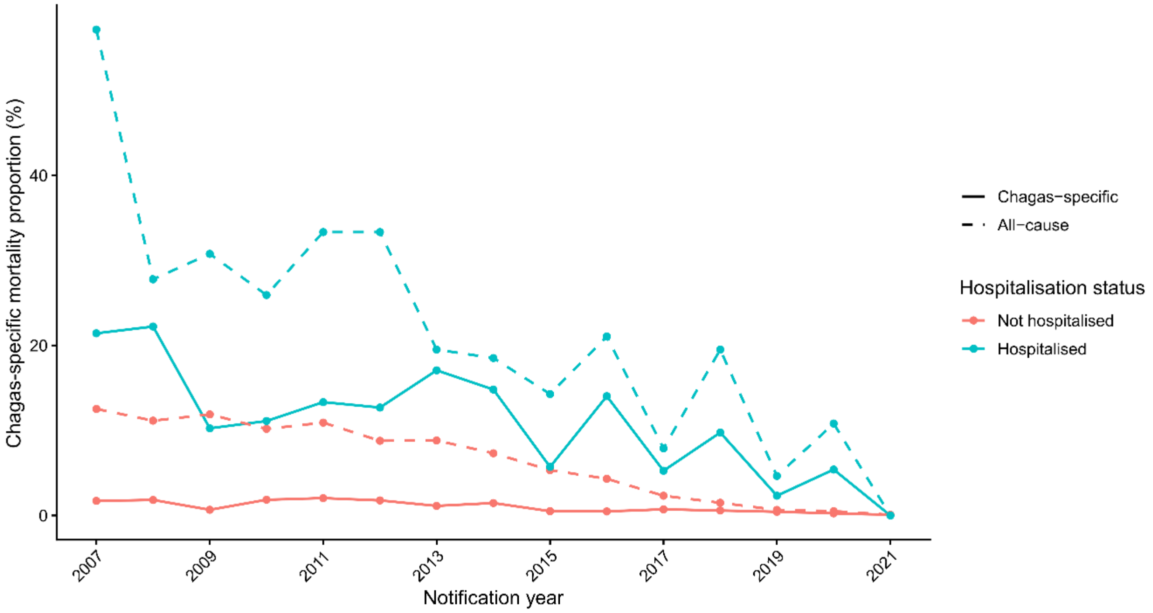

Note:

[a] Solid and dashed lines represent Chagas-specific and all-cause mortality proportion (%), respectively. Colours indicate hospitalisation status. Annual Chagas-specific and all-cause mortality proportion (%) are crude proportions calculated by notification year.

Supplementary Figure 7. Annual Chagas-specific and all-cause mortality proportion (%) by number of hospitalisations and notification year, Chile, 2007–2021.

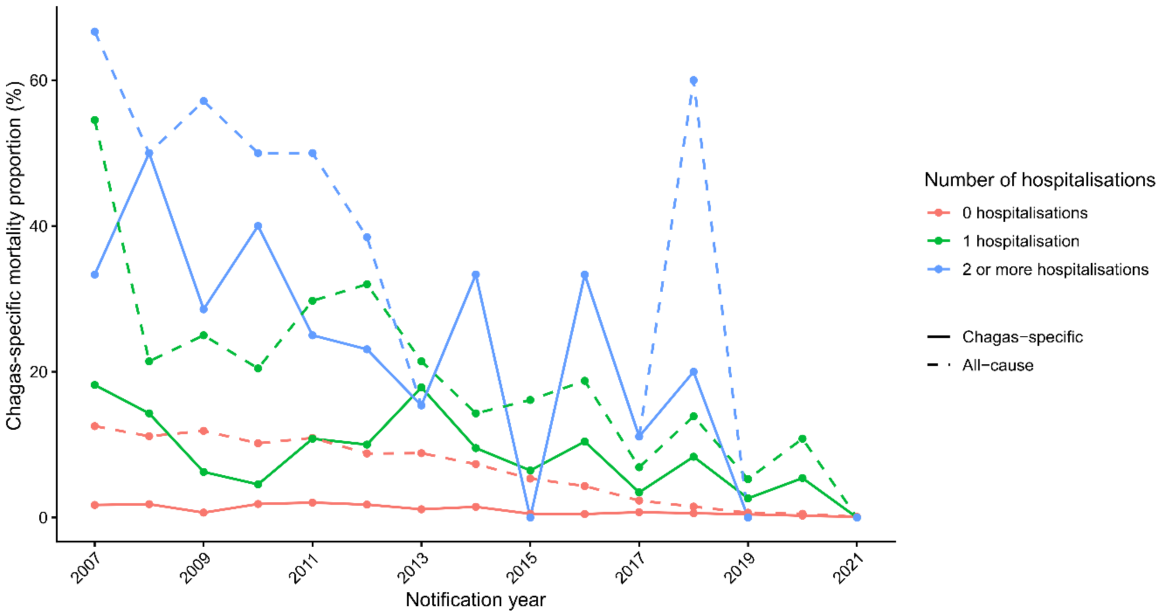

Note:

[a] Solid and dashed lines represent Chagas-specific and all-cause mortality proportion (%), respectively. Colours indicate hospitalisation status. Mortality proportion are crude proportions calculated by notification year.

Supplementary Figure 8. Annual Chagas-specific and all-cause mortality proportion (%) among hospitalised patients by sex and notification year, Chile, 2007–2021.

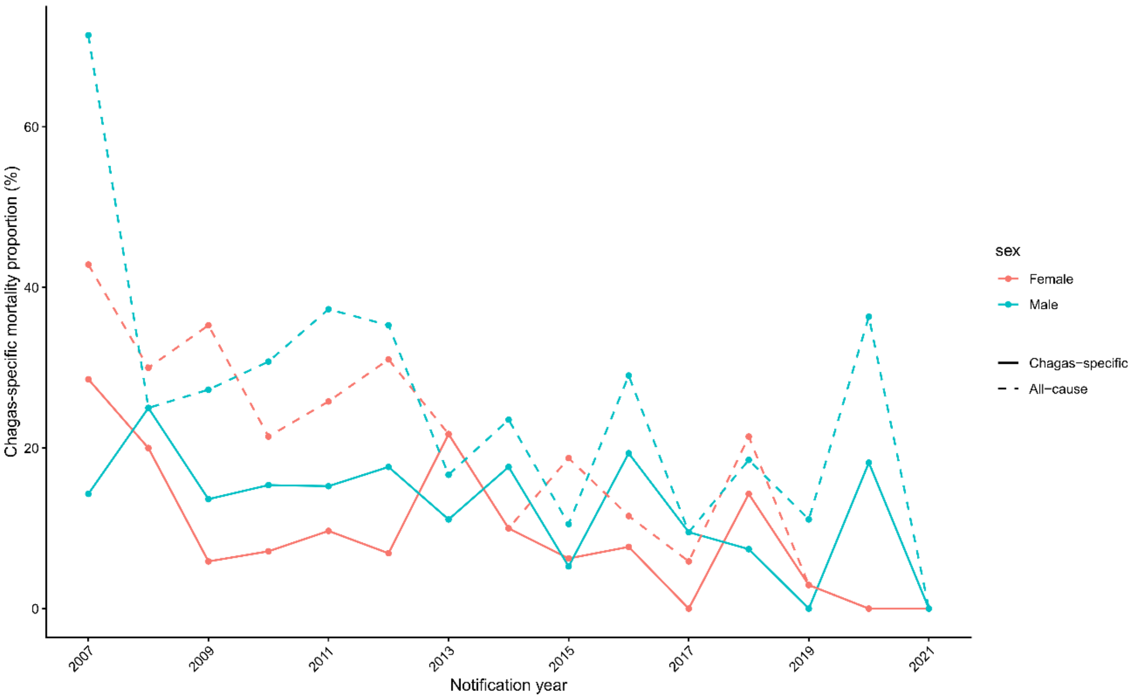

Note:

[a] Solid and dashed lines represent Chagas-specific and all-cause mortality proportion (%), respectively. Colours indicate hospitalisation status. Annual Chagas-specific and all-cause mortality proportions (%) are crude proportions calculated by notification year.

Supplementary Figure 9. Annual Chagas-specific and all-cause mortality proportion (%) among hospitalised patients by age and notification year, Chile, 2007–2021.

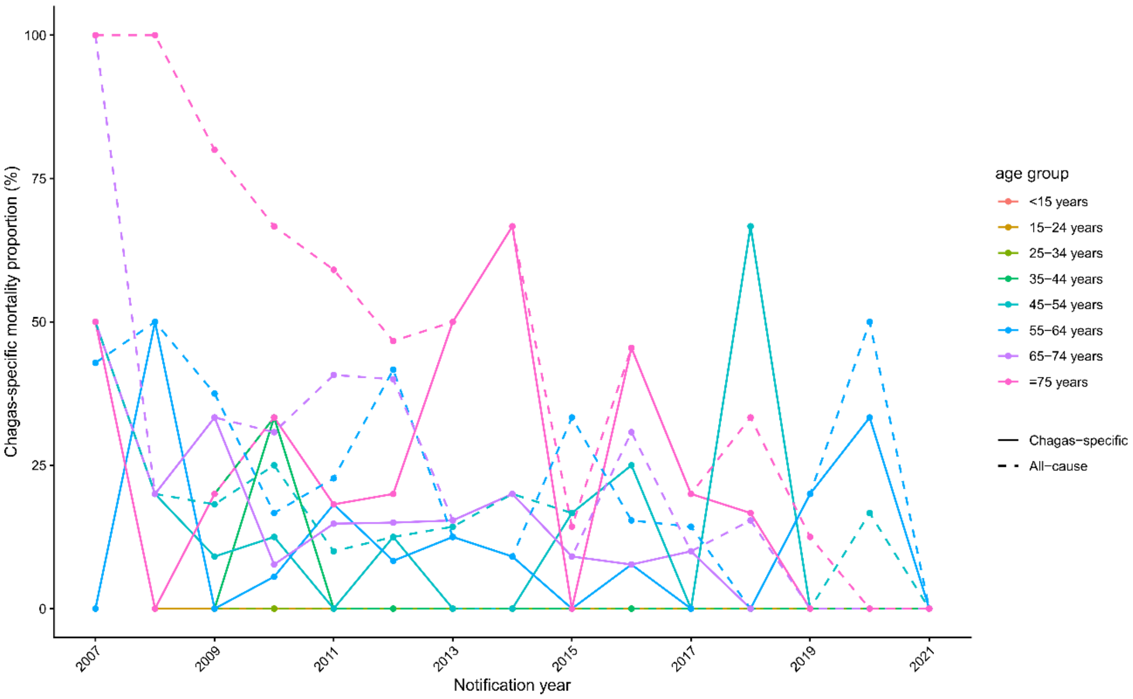

Note:

[a] Solid and dashed lines represent Chagas-specific and all-cause mortality proportion (%), respectively. Colours indicate hospitalisation status. Annual Chagas-specific and all-cause mortality proportions (%) are crude proportions calculated by notification year.

Supplementary Figure 10. Annual Chagas-specific and all-cause mortality proportion (%) among hospitalised patients by diagnostic stage and notification year, Chile, 2007–2021.

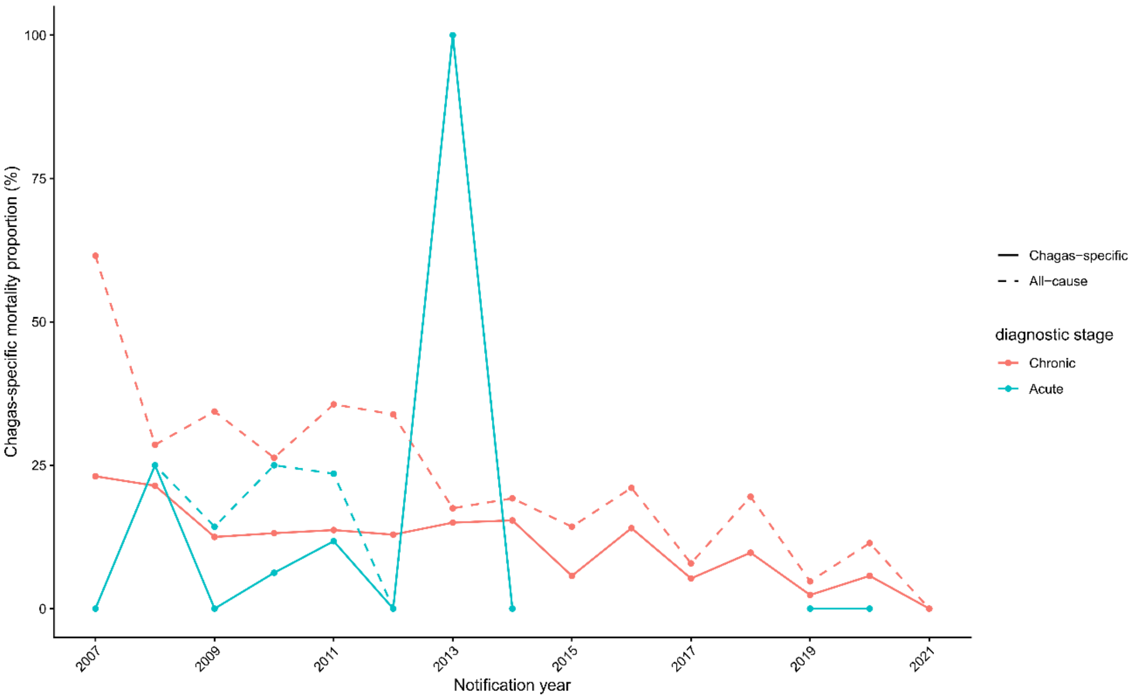

Note:

[a] Solid and dashed lines represent Chagas-specific and all-cause mortality proportion (%), respectively. Colours indicate hospitalisation status. Annual Chagas-specific and all-cause mortality proportions (%) are crude proportions calculated by notification year.

Supplementary Figure 11. Annual Chagas-specific and all-cause mortality proportion (%) among hospitalised patients by diagnosis and notification year, Chile, 2007–2021.

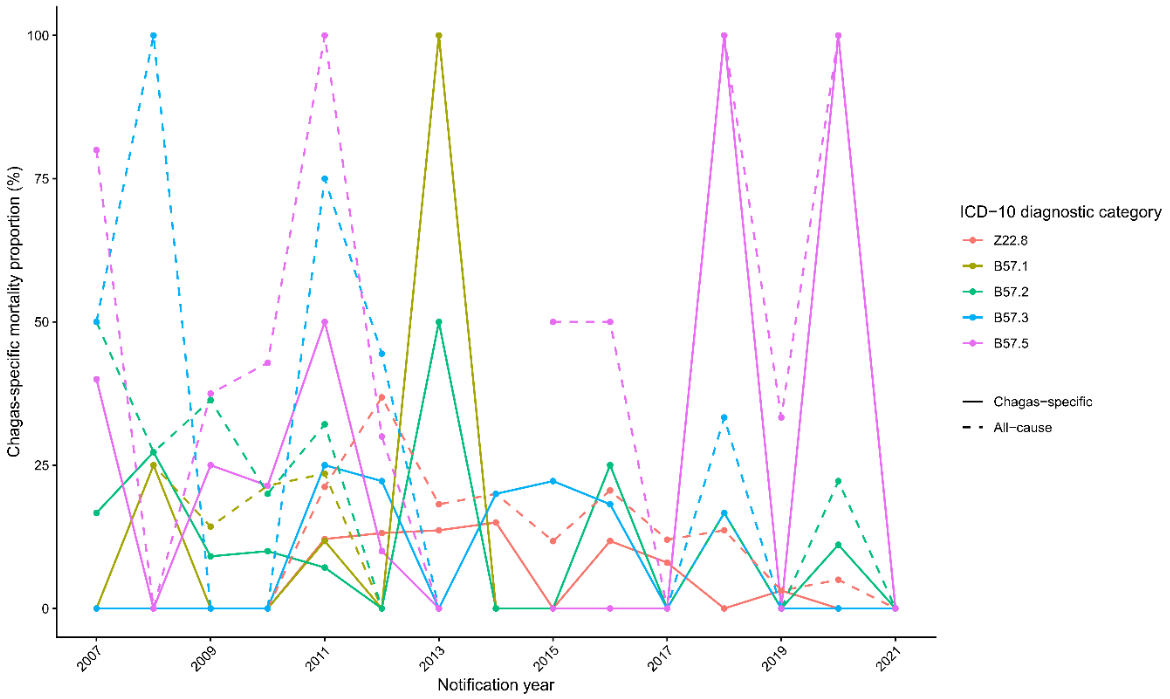

Note:

[a] Solid and dashed lines represent Chagas-specific and all-cause mortality proportion (%), respectively. Colours indicate hospitalisation status. Annual Chagas-specific and all-cause mortality proportions (%) are crude proportions calculated by notification year.

Supplementary Figure 12. Annual Chagas-specific and all-cause mortality proportion (%) among hospitalised patients by geographic zone and notification year, Chile, 2007–2021.

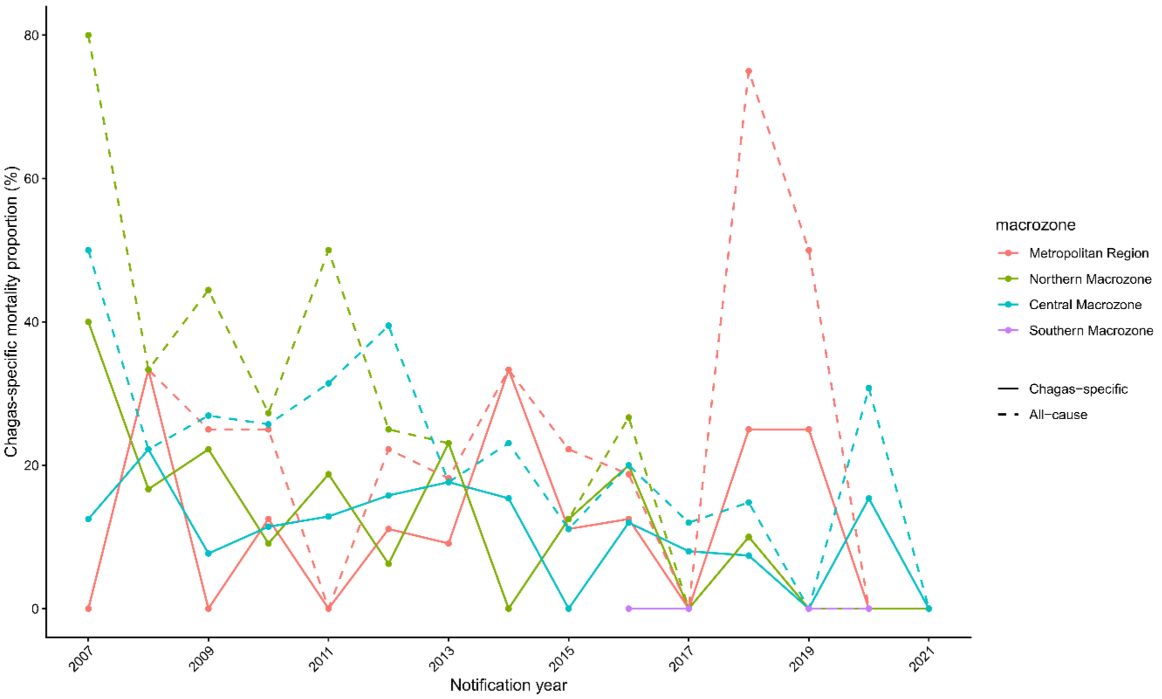

Note:

[a] Solid and dashed lines represent Chagas-specific and all-cause mortality proportion (%), respectively. Colours indicate hospitalisation status. Annual Chagas-specific and all-cause mortality proportions (%) are crude proportions calculated by notification year.

Supplementary Figure 13. Cumulative incidence functions for Chagas-specific and other-cause mortality in Chile, 2007–2021, stratified by age group

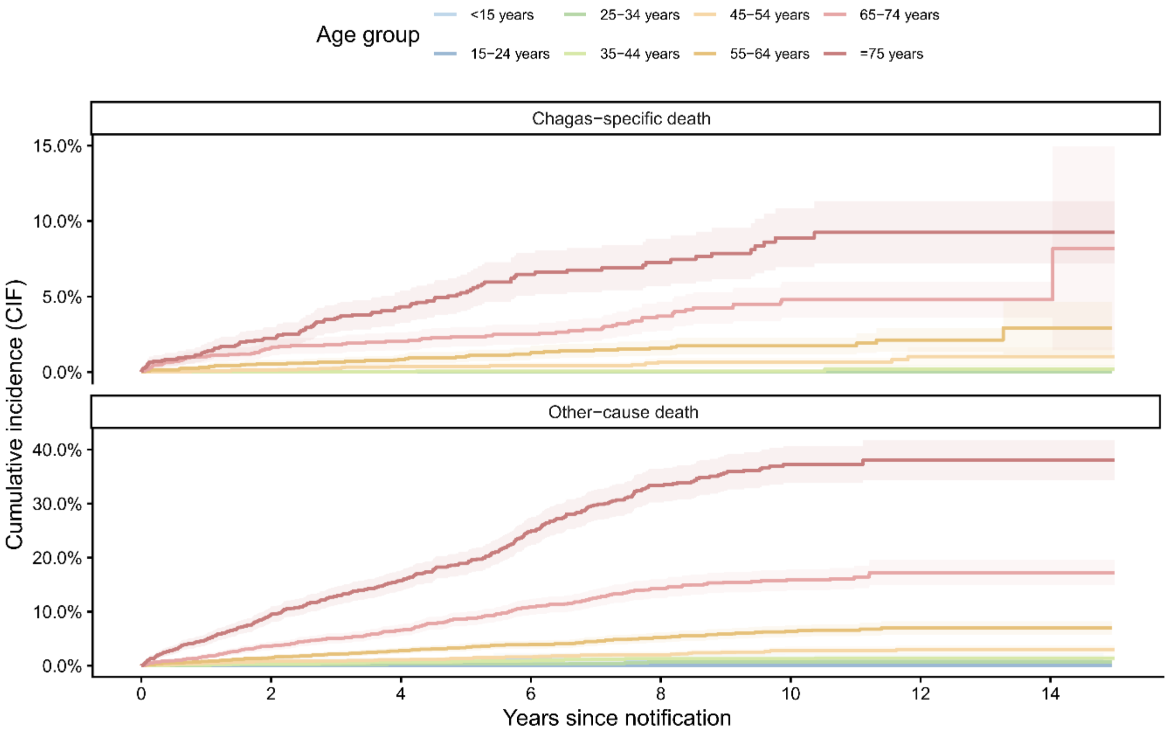

Notes

[a] The panels display cumulative incidence functions (CIF) for Chagas-specific death and other cause death from the date of notification, treating competing events.

[b] Gray's test p-values for between-group differences are reported within each panel (event 1: Chagas-specific death; event 2: other-cause death).

[c] Time is shown in years since notification; CIF is expressed as a percentage.

[d] A p-value <0.05 was considered statistically significant.

Supplementary Figure 14. Cumulative incidence functions for Chagas-specific and other-cause mortality in Chile, 2007–2021, stratified by sex.

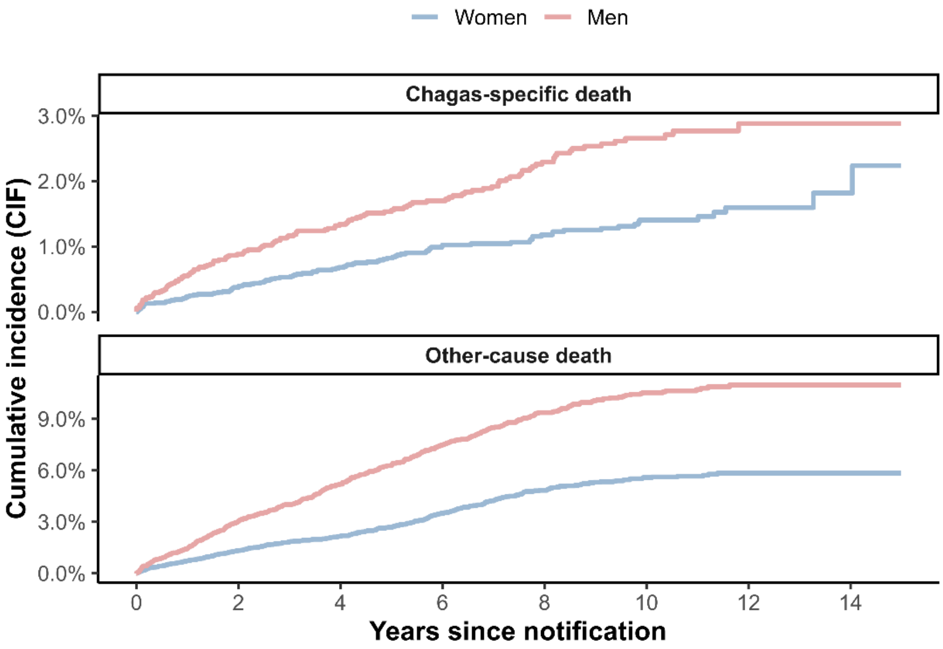

Notes

[a] The panels display cumulative incidence functions (CIF) for Chagas-specific death and other cause death from the date of notification, treating competing events.

[b] Gray's test p-values for between-group differences are reported within each panel (event 1: Chagas-specific death; event 2: other-cause death).

[c] Time is shown in years since notification; CIF is expressed as a percentage.

[d] A p-value <0.05 was considered statistically significant.

Supplementary Figure 15. Cumulative incidence functions for Chagas-specific and other-cause mortality in Chile, 2007–2021, stratified by diagnostic stage.

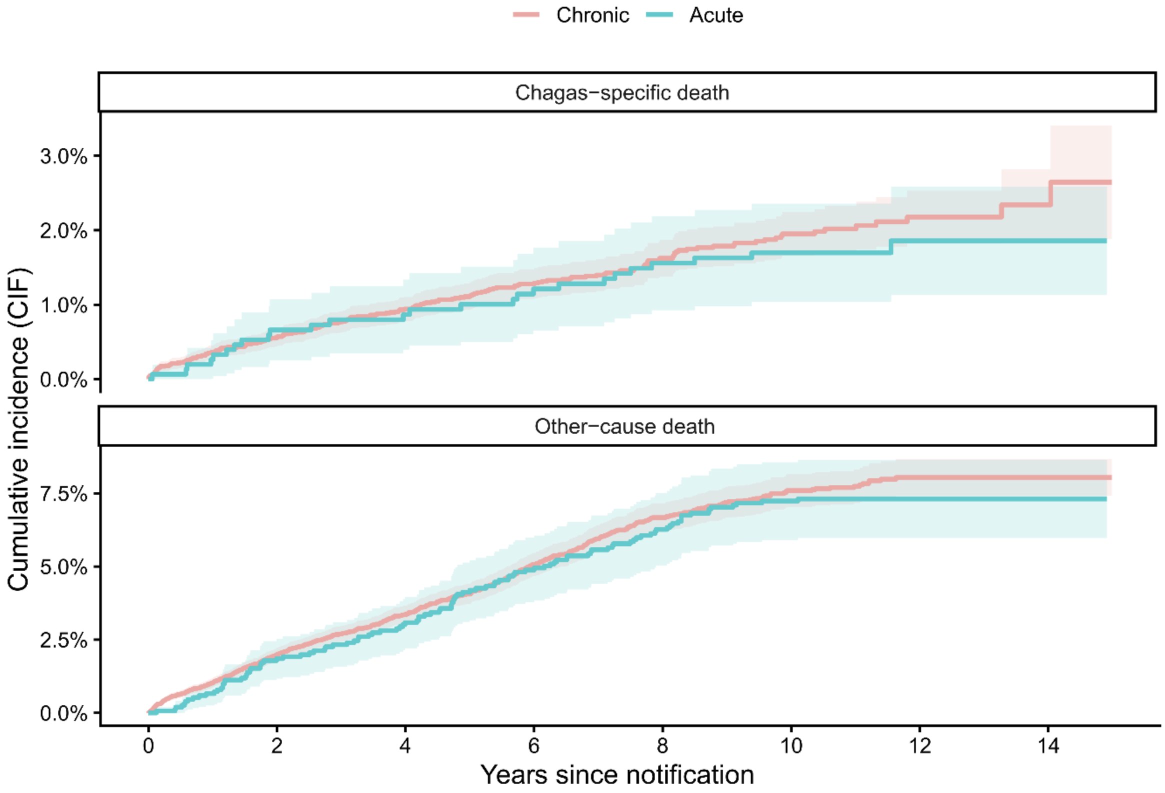

Notes

[a] The panels display cumulative incidence functions (CIF) for Chagas-specific death and other cause death from the date of notification, treating competing events.

[b] Gray's test p-values for between-group differences are reported within each panel (event 1: Chagas-specific death; event 2: other-cause death).

[c] Time is shown in years since notification; CIF is expressed as a percentage.

[d] A p-value <0.05 was considered statistically significant.

Supplementary Figure 16. Cumulative incidence functions for Chagas-specific and other-cause mortality in Chile, 2007–2021, stratified by ICD-10 codes.

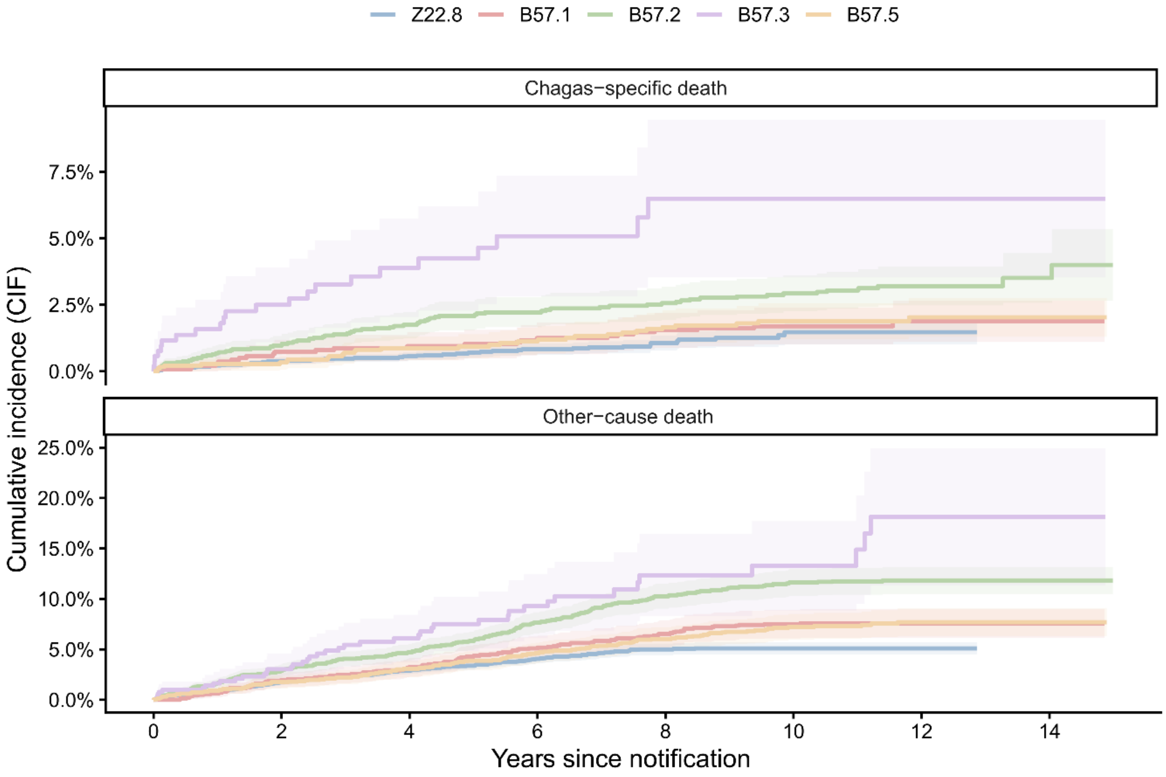

Notes

[a] The panels display cumulative incidence functions (CIF) for Chagas-specific death and other cause death from the date of notification, treating competing events.

[b] Gray's test p-values for between-group differences are reported within each panel (event 1: Chagas-specific death; event 2: other-cause death).

[c] Time is shown in years since notification; CIF is expressed as a percentage.

[d] A p-value <0.05 was considered statistically significant.

Supplementary Figure 17. Cumulative incidence functions for Chagas-specific and other-cause mortality in Chile, 2007–2021, stratified by geographic zone.

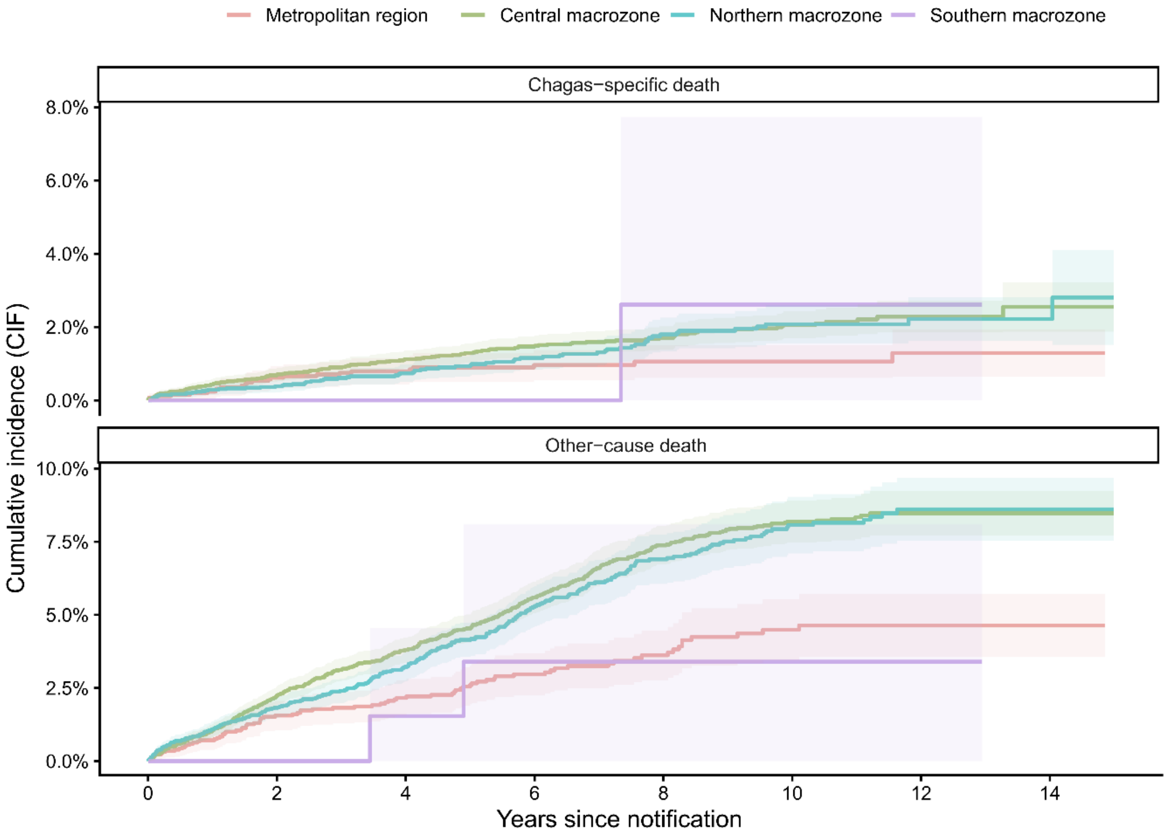

Notes

[a] The panels display cumulative incidence functions (CIF) for Chagas-specific death and other cause death from the date of notification, treating competing events.

[b] Gray's test p-values for between-group differences are reported within each panel (event 1: Chagas-specific death; event 2: other-cause death).

[c] Time is shown in years since notification; CIF is expressed as a percentage.

[d] A p-value <0.05 was considered statistically significant.
